# Chemical Reaction Networks Explain Gas Evolution Mechanisms in Mg-Ion Batteries

**DOI:** 10.1021/jacs.3c02222

**Published:** 2023-05-26

**Authors:** Evan Walter Clark Spotte-Smith, Samuel M. Blau, Daniel Barter, Noel J. Leon, Nathan T. Hahn, Nikita S. Redkar, Kevin R. Zavadil, Chen Liao, Kristin A. Persson

**Affiliations:** † Materials Science Division, Lawrence Berkeley National Laboratory, 1 Cyclotron Road, Berkeley, California 94720, United States; ‡ Department of Materials Science and Engineering, University of California, Berkeley, 210 Hearst Memorial Mining Building, Berkeley, California 94720, United States; § Energy Storage and Distributed Resources, Lawrence Berkeley National Laboratory, 1 Cyclotron Road, Berkeley, California 94720, United States; ∥ Argonne National Laboratory, 9700 South Cass Avenue, Lemont, Illinois 60439, United States; ⊥ Material, Physical and Chemical Sciences Center, Sandia National Laboratories, 1515 Eubank Boulevard SE, Albuquerque, New Mexico 87123, United States; # Department of Chemical and Biomolecular Engineering, University of California, Berkeley, 201 Gilman Hall, Berkeley, California 94720, United States; ∇ Molecular Foundry, Lawrence Berkeley National Laboratory, 1 Cyclotron Road, Berkeley, California 94720, United States

## Abstract

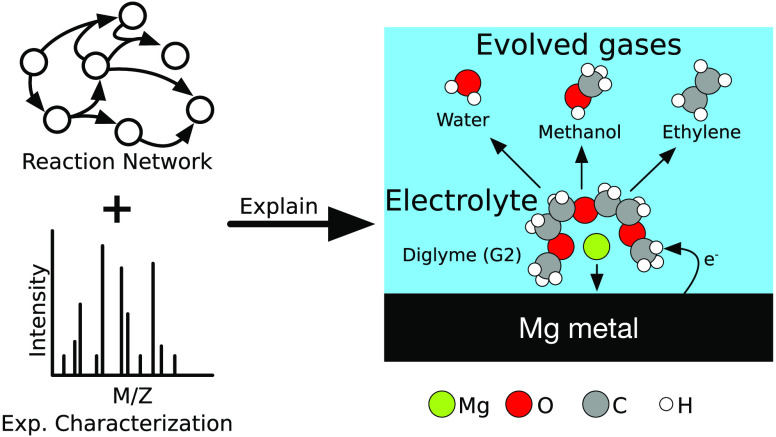

Out-of-equilibrium electrochemical reaction mechanisms are notoriously difficult to characterize. However, such reactions are critical for a range of technological applications. For instance, in metal-ion batteries, spontaneous electrolyte degradation controls electrode passivation and battery cycle life. Here, to improve our ability to elucidate electrochemical reactivity, we for the first time combine computational chemical reaction network (CRN) analysis based on density functional theory (DFT) and differential electrochemical mass spectroscopy (DEMS) to study gas evolution from a model Mg-ion battery electrolyte—magnesium bistriflimide (Mg(TFSI)_2_) dissolved in diglyme (G2). Automated CRN analysis allows for the facile interpretation of DEMS data, revealing H_2_O, C_2_H_4_, and CH_3_OH as major products of G2 decomposition. These findings are further explained by identifying elementary mechanisms using DFT. While TFSI^–^ is reactive at Mg electrodes, we find that it does not meaningfully contribute to gas evolution. The combined theoretical–experimental approach developed here provides a means to effectively predict electrolyte decomposition products and pathways when initially unknown.

## Introduction

Electrochemistry is increasingly applied to drive sustainable chemical and materials synthesis,^1,2^ efficiently process wastewater,^3^ and store renewable energy on the personal and the grid scale.^4,5^ The design of electrochemical technologies in these and other areas requires a deep understanding of reactivity at electrified interfaces. Unfortunately, such an understanding is notoriously elusive, particularly due to the essential role of complex, spontaneous cascade processes. There has been recent interest in applying high-throughput experimentation and machine learning to discover electrochemical reactions and predict reaction outcomes,^6,7^ yet the identification of electrochemical reaction pathways and products remains challenging.^8^ Electrochemical reaction mechanisms likewise cannot be easily analyzed by experiment,^9^ in part because they are frequently driven by short-lived radical and ionic intermediates.^10^

Electrolyte degradation in metal-ion batteries is an example of a technologically important and highly complex electrochemical reaction cascade. In lithium-ion batteries (LIBs), electrolytes decompose upon reduction to form solid electrolyte interphase (SEI) layers,^11,12^ which enable reversible lithium transport to and from the electrode while limiting or eliminating further electron transport to the electrolyte.

In order to meet growing global demand for energy storage while mitigating resource scarcity as well as geopolitical supply chain risk,^13,14^ alternative battery technologies are needed. Magnesium-ion batteries (MIBs) present one such possible beyond-Li-ion technology, alleviating some of the inherent limitations of current LIBs. However, the potential of MIBs is presently unrealized because of comparatively poor cycling behavior and unfavorable electrode passivation. Most electrolytes decompose at Mg negative electrodes during MIB charging. However, rather than forming effective SEI layers as in LIBs, many MIB electrolytes degrade to produce ionically insulating films, which prevent reversible Mg plating and stripping.^15,16^ In fact, it was once widely believed that all electrolyte decomposition at Mg electrodes would lead to electrochemically inert films,^17^ and the first instances of electrolytes decomposing to produce protective non-ionically insulating SEI films on Mg were only discovered in the past ten years.^18−20^

Previous studies have provided relatively little detail regarding either the reaction mechanisms or decomposition products involved in MIB electrolyte decomposition and interphase formation. In most cases where MIB interphases have been characterized,^18−24^ the techniques used have identified simple inorganic components (e.g., MgO, MgS, or MgCO_3_) or bonding motifs (e.g., C—O or C=O groups), unable to provide specific insight into organic speciation. Theoretical studies using density functional theory (DFT) and *ab initio* molecular dynamics (AIMD) can provide more detailed insight into electrolyte reactivity. However, previous DFT studies have primarily or exclusively considered the initial steps of electrolyte decomposition,^25−27^ while AIMD is generally limited to extremely short time scales (∼10 ps) at idealized interfaces.^21,28^

In this work, we conduct a combined theoretical–experimental analysis to probe electrolyte degradation and gas evolution in a model MIB electrolyte—magnesium bistriflimide (Mg(TFSI)_2_) dissolved in diglyme (G2). We perform online electrochemical mass spectroscopy (OEMS), a kind of differential electrochemical mass spectroscopy (DEMS), to detect gaseous byproducts of MIB electrolyte decomposition *in situ*. DEMS is a useful tool for instantaneous and quantitative detection of gaseous species evolved from solution during electrochemical testing,^29,30^ and it has previously been used to quantitatively diagnose the gaseous species generated during LIB cycling.^31−33^ However, DEMS has not been extensively applied to study gas evolution in MIBs. Due to the limited understanding of electrolyte decomposition in MIBs, spectroscopic interpretation for MIBs is more challenging than that for LIBs.

Computational modeling can aid in the interpretation of the experimental spectra. In particular, chemical reaction networks (CRNs) are natural tools for combined theoretical–experimental studies, as they can be applied to identify important species in a reactive system and even study reactive dynamics.^34^ We recently developed a general CRN methodology^35^ to automatically predict CRN products, as well as reaction pathways to form those products. Here, for the first time, we combine this platform with experimental characterization techniques to understand reactivity in batteries. We construct the first ever CRN describing MIB electrolyte decomposition and SEI formation at the Mg plating potential. By screening the predicted products of this CRN by their calculated liquid–gas solubility, we are able to identify possible evolved gases and from these positively identify the gases observed experimentally in OEMS. Analyzing elementary reaction mechanisms for the formation of these possible gases, we explain why some gases form while others do not. Our approach of combining CRN analysis with experimental spectroscopy provides a path forward for the in-depth analysis of chemical transformations in next-generation electrochemical systems with minimal prior knowledge.

## Computational Methods

### Species and Molecular Property Data Set

A data set of species relevant to Mg(TFSI)_2_/G2 electrolyte decomposition and interphase formation, the **MA**gnesium **D**ataset of **E**lectrolyte and **I**nterphase **R**e**A**gents (MADEIRA), was constructed using high-throughput DFT. The approach taken for the construction of this data set was similar to that used to develop the lithium-ion battery electrolyte (LIBE) data set reported previously.^36^ Electrolyte species (including G2, TFSI^–^, and related complexes with Mg ions) and known or suspected products were broken down into a set of fragment molecules. Due to limited experimental characterization, the products included were only inorganic species (e.g., MgSO_3_) and small molecule gases (e.g., H_2_). For each fragment, we obtained an optimized geometry, Gibbs free energy, and other properties (including atomic partial charges and atomic partial spin) using DFT with the ωB97X-V density functional,^37^ def2-TZVPPD basis set,^38^ and solvent model with density (SMD)^39^ with solvent parameters for G2.^40^ We denote this level of theory as ωB97X-V/def2-TZVPPD/SMD(G2). Additional species were included based on selective recombination of the fragments. All calculations were conducted using the Q-Chem electronic structure code, version 5,^41^ and calculations were conducted in high throughput using the atomate(42) and custodian(43,44) libraries.

The complete data set obtained using this procedure is available on Figshare.^45^ We note that, because few products—and essentially no organic or polymeric products—of Mg(TFSI)_2_/G2 electrolyte decomposition have been positively identified, we were not able to use knowledge of such products to improve the coverage of the data set. As a result, the set of species obtained by this fragmentation–recombination procedure is almost certainly incomplete, with key species relevant to electrolyte decomposition and SEI formation likely missing. Work to expand this data set is ongoing. We also note that we intend to describe this data set in further detail in a future publication.

## CRN Generation

### Solvation Correction

While implicit solvation methods such as SMD are suitable for solution-phase calculations involving neutral and charged organic species, they severely underestimate the stabilizing effect of solvent on metal ions.^35^

To correct the (free) energies of species with undercoordinated Mg ions in our reaction network, we calculated the average effect of each coordinate bond on the Mg^2+^ and Mg^1+^ ions. We optimized Mg^2+^(G_2_)_*n*_ and Mg^1+^(G_2_)_*n*_ clusters using DFT in Q-Chem, with *n* ∈ {0, 1, 2}. To lower the cost of these calculations, we optimized the clusters at the ωB97X-D/def2-SVPD/PCM^38,46,47^ (ε = 7.23) level of theory, with single-point energy corrections performed at the ωB97X-V/def2-TZVPPD/SMD(G2) level of theory as described above. We found (Supporting Information Figure S13) that each Mg–O coordinate bond stabilized Mg^2+^ by 1.37 eV, while Mg^1+^ was stabilized by 0.49 eV for each coordinate bond. In network construction, these values were modified slightly to 1.49 and 0.56 eV, respectively, in order to make the expected coordination reactions slightly exergonic.

If any Mg ions are undercoordinated, then the free energy is lowered by the correction factors for each “missing” coordinate bond. We use partial charges obtained from Natural Bonding Orbital (NBO) version 5.0^48^ analysis to determine the charge state of each Mg ion in order to apply the appropriate correction. When determining the number of “missing coordinate bonds”, we assume that Mg^2+^ generally prefers a 6-fold coordination and Mg^1+^ prefers a 5-fold coordination.

As in our previous study,^35^ when calculating reaction free energies for oxidation or reduction reactions, we used an uncorrected free energy. This is especially important for reduction reactions involving Mg due to the different preferred coordination environments of Mg^2+^ and Mg^1+^. In addition, we do not apply a solvation correction when calculating the energy barriers. The assumptions implicit in performing a correction for metal-ion solvation, namely, that the ion is always in an equilibrium solvation structure, break down when considering transition states, which are inherently nonequilibrium structures.

### Species Filtering

We used the high-performance reaction generation (HiPRGen) method^35^ to automatically construct CRNs from an initial set of species and their properties. HiPRGen is designed for cases where potential energy surface (PES) exploration techniques (stochastic surface walking,^49^ AIMD, etc.) are too expensive to thoroughly capture the reactivity of a system and for which reaction patterns are not sufficiently well understood to allow the use of prescriptive reaction templates. HiPRGen has previously been used to construct and analyze CRNs relevant to electrolyte degradation and SEI formation in LIBs^35,50^ but has not previously been applied to study MIBs.

Instead of using PES exploration or templates, HiPRGen constructs CRNs by using extensible filters. For this work, the following types of species were excluded:

- Molecules containing neutral or negative metal ions, where the charges are calculating by applying NBO to a single-point energy calculation at the ωB97X-V/def2-TZVPPD/SMD(G2) level of theory
- Molecules composed of two or more disconnected fragments
- Metal-centric complexes, where two or more nonmetal fragments are connected only by coordinate bonds to Mg ions
- Molecules with a charge less than −2 or greater than 2

In addition to these filters, we ensure that there are no redundant species. That is, if multiple molecules exist with the same charge, spin multiplicity, and structure (neglecting coordinate bonds with metal ions), then we include only the molecule with the lowest solvation-corrected free energy. Using these filters, an initial set of 11,502 species was reduced to 6,469 species.

### Reaction Filtering

After the species have been filtered, HiPRGen enumerates all possible stoichiometrically valid unimolecular or bimolecular reactions between these species. Because we are interested in electrochemical processes, where the electrolyte system is open to electrons, these stoichiometrically valid reactions conserve mass but do not necessarily conserve charge. Then, the stoichiometrically valid reactions are filtered in much the same way as the species are filtered. For this work, we used the same set of reaction filters that we previously reported.^35^ As some examples, we remove:

- Endergonic reactions with Δ*G* > 0 eV
- Reduction or oxidation reactions involving more than one electron (|Δ*q*| > 1)
- Reactions involving spectators that do not directly participate
- Reactions involving more than two covalent bonds changing simultaneously

In total, we obtained 92,812,997 unique reactions using this filtering procedure.

### Identification of CRN Products

We employed the Gillespie algorithm,^51,52^ a stochastic method, to sample the reactive space defined by the HiPRGen-generated CRN. In order to explore as many diverse reaction pathways as possible, we conducted simulations with various initial states:

- 30 Mg^2+^, 30 G2, and 30 TFSI^–^
- 30 Mg^2+^, 30 G2, 30 TFSI^–^, and 30 CO_2_
- 30 Mg^2+^, 30 G2, 30 TFSI^–^, and 30 OH^–^
- 30 Mg^2+^, 30 G2, 30 TFSI^–^, 30 OH^–^, and 30 H^•^
- 30 Mg^2+^, 30 G2, 30 TFSI^–^, 30 CO_2_, 30 OH^–^, and 30 H^•^

The choice to include 30 of each initial species is arbitrary and was determined empirically. Simulations involving too few molecules in the initial state will not allow many reactions to be sampled, while simulations involving many molecules will complete more slowly.

For each initial state, 50,000 trajectories of at most 250 steps were conducted. For each of the five sets of simulations, we obtained stepwise average trajectories. The smoothing of the average trajectories (Supporting Information Figures S8–S12) indicates convergence to the exact expected behavior and confirms that we have sampled sufficiently. All simulations were conducted at the equilibrium potential of Mg (0 V vs Mg/Mg^2+^).

Using the average trajectories, we automatically identified the CRN products. These CRN products are not necessarily the products of the corresponding real chemical system, but we have previously found^35^ significant overlap between CRN products and experimentally observed products in battery electrolyte systems. CRN products are defined using three heuristics previously described by Barter, Spotte-Smith, et al.^35^ Specifically, a CRN product has a formation:consumption ratio of at least 1.5 (the species must be formed three times as a product of a reaction for every two times it is consumed as a reactant), has an average amount of at least 0.1 in the final state (at least one of the species remains at the end of every ten trajectories), and can be formed via a pathway with a cost lower than 10, where the cost of a reaction is Φ = exp(Δ*G*/*k*_B_*T*) + 1 and the cost of a pathway is the sum of the costs of the elementary steps involved. We further remove CRN products that are open-shell, as we generally believe that radical species should be short-lived. The CRN products vary depending on the initial conditions. A description of all predicted CRN products can be found in the Supporting Information (see Figures S14–S16).

### Discovery of Elementary Reaction Mechanisms

We identified elementary reaction mechanisms using the AutoTS workflow,^53^ which is powered by Jaguar’s electronic structure code.^54^ All initial transition state searches were conducted using the ωB97X-D density functional with the def2-SVPD(-f) basis set and the PCM implicit solvent model with water as a solvent. A single-point energy correction was then applied by using the ωB97M-V functional^55^ with a larger def2-TZVPD basis set and the PCM implicit solvent model. We note that ωB97M-V is exceptionally accurate for calculations of the reaction energy barriers and reaction thermodynamics.^56^ All transition states were validated by confirming that they connect the expected reaction end points. All energy barriers reported in this work are based on an infinite-separation approximation; that is, the free energies of reaction reactants and products are calculated from the free energies of individual isolated species rather than reaction entrance or exit complexes.

### Calculation of Reduction Potentials

When constructing and analyzing CRNs, we intentionally remove clusters with multiple molecules bound to Mg ions (see Species Filtering above). In part, this is necessary in order to limit the size of the CRN. However, this means that essentially all Mg ions in our data set are undercoordinated. As we note (see Solvation Correction), for chemical reactions, we can account for this undercoordination via a simple linear correction to the free energy, but the same correction cannot easily be applied to reduction reactions, especially if Mg ions are being reduced.

Here we report reduction potentials based on calculations in an implicit solvent at the ωB97X-D/def2-SVPD(-f)/PCM//ωB97M-V/def2-TZVPD/PCM level of theory. From the Gibbs free energies of the reduced and nonreduced species, the reduction potential is calculated as
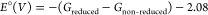
1where the Gibbs free energies are reported in eV and the shift by 2.08 V is necessary in order to report potentials referenced to a Mg/Mg^2+^ electrode. In the Supporting Information (Table S1), we also calculate reduction potentials where Mg ions are fully solvated by an explicit solvent shell.

### Estimation of Solubility in Diglyme

We calculate the liquid–vapor solubility limits of CRN products in G2 *S*_G2_ via
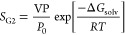
2where VP is the vapor pressure of the solute (in atmospheres or atm), *P*_0_ is the pressure of a standard-state (1 M) ideal gas at room temperature (24.45 atm), *R* is the ideal gas constant (8.314 J mol^–1^ K^–1^), *T* is the absolute temperature (298.15 K for room temperature), and Δ*G*_solv_ is the free energy of solvation. This equation assumes that the solutes of interest behave ideally in both the gas and solution phase. We also neglected the effect of the dissolved salt in G2 and treated the solvent as a pure organic liquid. We predict the vapor pressure of CRN products using the SIMPOL^57^ group contribution method (as implemented in UManSysProp),^58^ and we calculate the free energy of solvation using SMD (specifically, via DFT calculations at the ωB97X-V/def2-TZVPPD/SMD(G2) level of theory). Because SIMPOL is specifically designed for multifunctional organic compounds, we instead provide experimental vapor pressures at room temperature for H_2_ and H_2_O.

We note that *ab initio* prediction of gas solubility limits is deeply challenging and an area of ongoing research. The method employed here was chosen for its ease and simplicity rather than for its accuracy. While we believe it is sufficiently accurate to distinguish between species that should or should not evolve as gases from an electrolyte, we do not expect quantitatively accurate predictions of solubility limits.

## Experimental Methods

### Electrolyte Preparation

All reagents and solvents were prepared using a Schlenk line or glovebox (with <1 ppm of O_2_ and <1 ppm of H_2_O) under an argon atmosphere. Mg(TFSI)_2_ (99.5%, Solvionic) was dried under a vacuum at 170 °C for 24–48 h prior to use. G2 (anhydrous, 99%, Sigma-Aldrich) was distilled over calcium hydride and stored on 3 and 4 Å molecular sieves. The distilled G2 had a water content of <5 ppm of H_2_O as measured by a Karl Fischer Coulometer Titrator. Mg(TFSI)_2_/G2 solutions were prepared in a glovebox with a volumetric flask charged with the appropriate amount of predried Mg(TFSI)_2_ powder dissolved in distilled G2 solvent.

### Online Electrochemical Mass Spectrometry

#### Device Configuration

Online electrochemical mass spectrometry (OEMS), one category of the differential electrochemical mass spectrometry (DEMS), was used for the instaneous and quantitative analysis of the gaseous species generated during electrochemical experiments. A schematic of our OEMS experimental setup is provided in the Supporting Information (Figure S1).

In our work, a modified capillary OEMS was used, which consisted of a supporting inert gas as a flow carrier (He) and a capillary inlet for mass spectroscopy. It has a moderate response time of 16 s, and the flow rate was controlled at ∼20 μL/min by a flow meter. Other features in our OEMS include (1) the ability to evacuate and flush the system with He after the DEMS cell was assembled inside the glovebox, (2) calibration to quantify the gaseous generation amount in real time, and (3) a flow system to enable detection of a small amount of gas production.

An FMA-2600/FVL-2600 SERIES Mass and Volumetric instrument from OMEGA was used to control the flow rate of a He tank. The Hiden HPR-40 DEMS system was equipped with a quadrupole mass spectrometer and a QIC UF microflow capillary inlet (type 303452) with a flow rate of 12 μL/min. A PX409-015GUSBH (Pressure Sensor, 15 psi, Digital, Gauge, 1/16 in.) transducer was used to measure the real-time pressure in order to quantify gaseous species. A total of five manual Swagelok ball valves (SS-41GS1) were incorporated into the system to allow evacuation of the gas line and control of the flow rate/testing. An ECC-DEMS cell from El-cell was used.

#### Electrochemical Measurements

A two-electrode setup was used for the experiments, with polished Mg metal as the counter electrode (CE) and reference electrode (RE) and a gold disc (Φ = 8 mm, Au, Aldrich, 99.99%, 0.1 mm thick) as the working electrode (WE). We note that Au can alloy with Mg, but under typical electrochemical experimental conditions, the extent of alloying is minimal, with only nanoscale alloy regions.^59^ We therefore expect that Au will not significantly affect the electrochemistry and reactivity of plated Mg.

#### OEMS Calibration

A calibration and conversion are required in order to report OEMS measured intensities in terms of either partial pressure or molar flow. The relative signal intensity of a species with mass-to-charge ratio *M*/*Z* (*x*_*M*/*Z*_) is calculated as
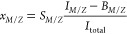
3where *S*_*M*/*Z*_ is a machine-specific sensitivity factor, *B*_*M*/*Z*_ is the background intensity, *I*_*M*/*Z*_ is the measured intensity at the mass-to-charge ratio of interest, and *I*_total_ is the total measured intensity.

Using the cell pressure *P*_total_, the relative signal intensity *x*_*M*/*Z*_ can be converted to a partial pressure
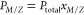
4From there, the quantity of gas detected (in moles) can be obtained using the ideal gas law
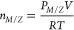
5where *R* is the ideal gas constant, *T* is the absolute temperature in Kelvin, and *V* is the head space volume in the DEMS cell.

### X-ray Photoelectron Spectroscopy and Scanning Electron Microscopy

Mg cycling and deposition for *ex situ* analyses were performed on planar Pt(111) textured substrates in a custom-built Teflon cell containing a Mg rod CE, Mg wire RE, and a WE area of 0.2 cm^2^. These substrates were prepared by evaporation of the noble metal onto Ti-coated Si wafers and were cleaned prior to use with acetone, 3:1 H_2_SO_4_:H_2_O_2_ (piranha solution), and deionized water, successively. Deposited Mg films were successively rinsed in G2 and 1,2-dimethoxyethane. Samples were transferred for X-ray photoelectron spectroscopy (XPS) using an inert transfer capsule. XPS was performed on a Kratos Axis Ultra spectrometer using a monochromatic Al Kα source. Analyses were performed on films after 10 s of Ar^+^ sputtering, and quantification was performed using CasaXPS software. SEM was performed on an FEI Magellan microscope.

## Results and Discussion

### OEMS

**Figure 1:**
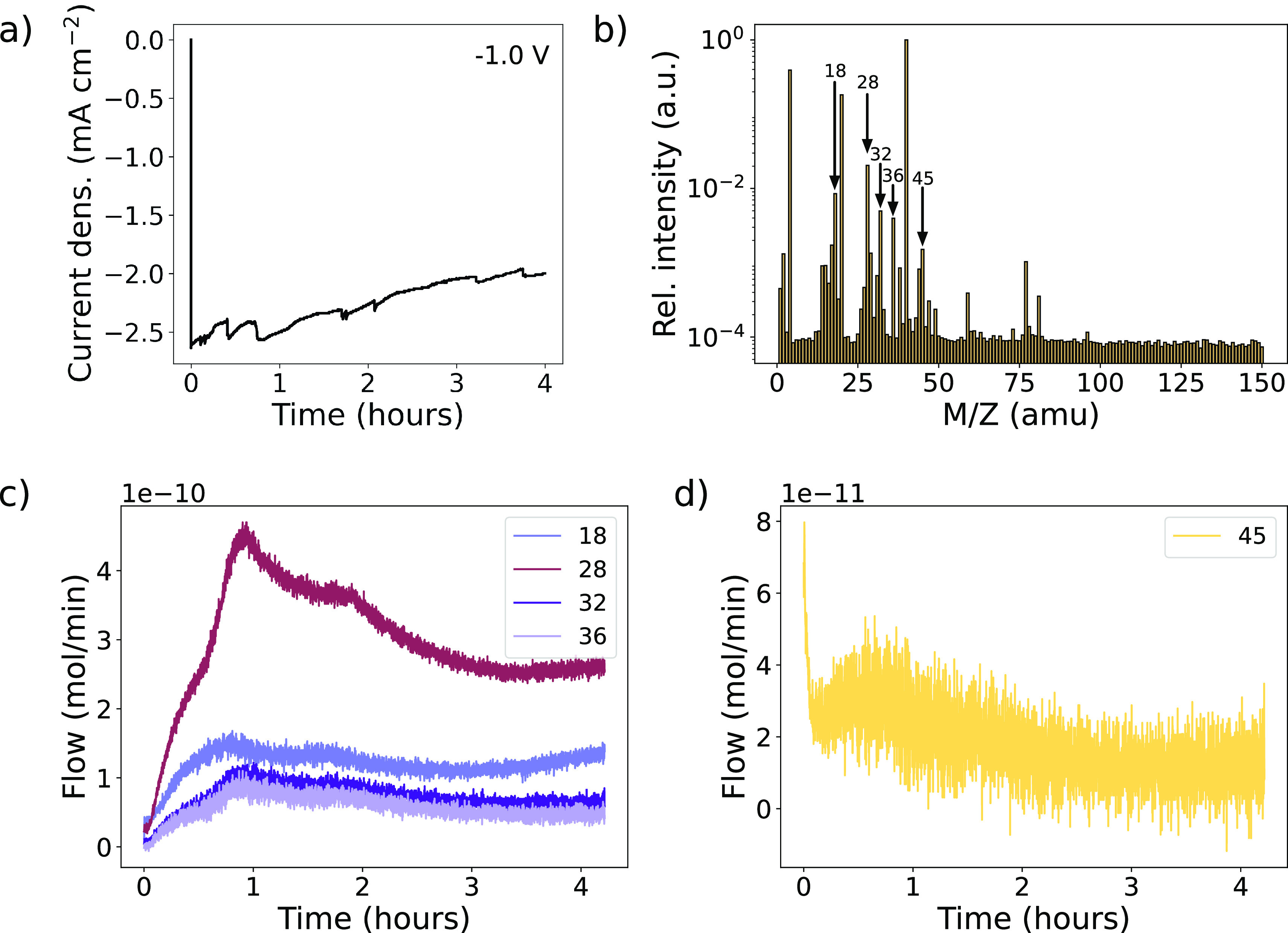
OEMS measurements on a Mg(TFSI)_2_/G2 electrolyte during a potentiostatic hold at a cell potential of −1.0 V. (a) The applied current density during potentiostatic hold; (b) integrated relative OEMS intensity (in log scale) after approximately 4 h of measurement, with major peaks indicated; (c) time-resolved flow for several major peaks (*M*/*Z* = 18, 28, 32, 36) demonstrating continuous evolution; (d) time-resolved flow for *M*/*Z* = 45 with initially high partial pressure that rapidly decays.

A 0.5 M Mg(TFSI)_2_/G2 electrolyte was used to plate Mg onto Au at a cell voltage of −1.0 V for approximately 4 h in the OEMS system described above. The electrochemical and OEMS measurements are shown in Figure 1; cyclic voltammetry data are shown in Supporting Information Figure S2. The current density during the potentiostatic hold (Figure 1a) is initially high (−2.65 mA/cm^2^) but gradually decreases in magnitude over time as a result of the increased resistance caused by electrode passivation. The dynamic resistance of the electrode interfaces is also evident from the sudden changes in current which occur at varying intervals.

The OEMS signal over the course of the experiment was integrated in order to identify the major peaks (Figure 1b; snapshot OEMS spectra are presented in Supporting Information Figures S3–S7). We ignore peaks at *M*/*Z* = 2, 4, 20, and 40, as these correspond to the carrier gas (He, *M*/*Z* = 2, 4) or Ar (*M*/*Z* = 20, 40) that was trapped in the Mg(TFSI)_2_/G2 electrolyte after the electrolyte was distilled and the DEMS cell was assembled in an Ar-filled glovebox. Other major peaks include those at *M*/*Z* = 18, 28, 32, 36, and 45.

From the time-resolved measurements (Figure 1c), we find that the signal at *M*/*Z* = 18 is relatively stable after an initial increase, while the signals at *M*/*Z* = 28, 32, and 36 all reach a maximum at ∼1 h and afterward gradually decrease. In contrast (Figure 1d), the *M*/*Z* = 45 flow rapidly decays to a near-zero signal in the first few minutes of the experiment.

This initial difference in signal over time suggests that the species detected at *M*/*Z* = 18, 28, 32, and 36 are products of ongoing reactivity, while the species detected at *M*/*Z* = 45 either is a decomposition product that can only form under highly specific conditions or is not indicative of a decomposition product at all. Given that this OEMS experiment was conducted in a constant-potential regime in which Mg is consistently plated (see Supporting Information Figure S2 for evidence of plating), we believe that the latter possibility is more likely. We suggest that the *M*/*Z* = 45 signal is likely indicative of G2 itself rather than a product of G2 decomposition formed at the Mg electrode. The initially high *M*/*Z* = 45 signal reflects evaporated G2 that built up in the DEMS cell during preparation; after this initial G2 is purged, evaporation continues slowly, resulting in a lower signal during the remainder of the experiment. We note that OEMS is typically not sufficiently specific to allow for positive identification of specific gases or molecular fragments. *M*/*Z* = 28, for instance, could indicate diatomic nitrogen (N_2_, M = 28 amu), carbon monoxide (CO, M = 28 amu), or ethylene (C_2_H_4_, M = 28 amu), among other possibilities.

### Identification of Observed Gases

In order to determine the identity of the major observed species, we constructed a CRN containing species that could be relevant to the decomposition of Mg(TFSI)_2_/G2 electrolytes and the subsequent interphase formation. Using stochastic simulations under five different initial conditions (see Identification of CRN Products), we identified 85 of an initial 6,469 species as CRN products (see the Supporting Information for more discussion). We believe that most electrolyte decomposition products will either precipitate and contribute to an interphase layer or otherwise be soluble in the electrolyte. Therefore, we filtered the predicted CRN products by their predicted solubility in G2 (*S*_G2_), using eq 2. Expecting considerable error in the prediction of *S*_G2_, we remove any predicted CRN product with a predicted solubility >5 M. We also remove ionic CRN products and CRN products containing Mg, as we expect such species to be considerably more stable in solution than in the gas phase.

**Figure 2:**
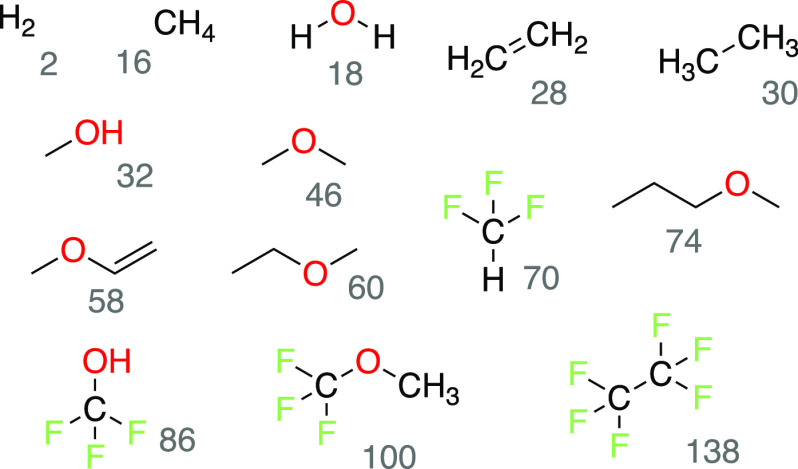
Gases predicted to evolve from Mg(TFSI)_2_/G2 electrolytes based on CRN analysis and prediction of solubility *S*_G2_. The mass of each CRN product (rounded, in amu) is shown next to the 2D structure in gray.

With these criteria, we predict that 14 of the 85 CRN products could evolve out of the solution and be detected by OEMS (Figure 2). These predicted gaseous CRN products enable the unambiguous assignment of the major observed OEMS peaks. For most peaks, there is exactly one gas that would be consistent with the signal. Specifically, the *M*/*Z* = 18 peak can be assigned to water (H_2_O), the *M*/*Z* = 28 peak can be assigned to ethylene (C_2_H_4_), and the *M*/*Z* = 32 peak can be assigned to methanol (CH_3_OH).

Notably, there are no predicted gaseous CRN products with masses consistent with *M*/*Z* = 36 and *M*/*Z* = 45 (although several species could produce fragments with *M* = 45 amu). This supports our previous suggestion that the *M*/*Z* = 45 signal does not correspond to a decomposition product but instead comes from another source, such as evaporated G2. We further suggest that the *M*/*Z* = 36 signal corresponds to an impurity species, rather than a decomposition product of either G2 or TFSI^–^. Considering that chloride (Cl^–^) is an impurity in commercial Mg(TFSI)_2_,^60^ we tentatively assign the *M*/*Z* = 36 peak to hydrogen chloride (HCl). This assignment is also consistent with the presence of a minor *M*/*Z* = 38 signal. The ratio of the integrated *M*/*Z* = 36 signal and the *M*/*Z* = 38 signal is 4.67, which is close to the ratio of the natural abundances of ^35^Cl to ^37^Cl (3.17).^61^

### Validating Predicted Major Products

To confirm that the peak assignments based on CRN products are reasonable, we identified formation pathways to several CRN products using the previously constructed CRNs and then used DFT to construct the elementary reaction mechanisms.

**Figure 3:**
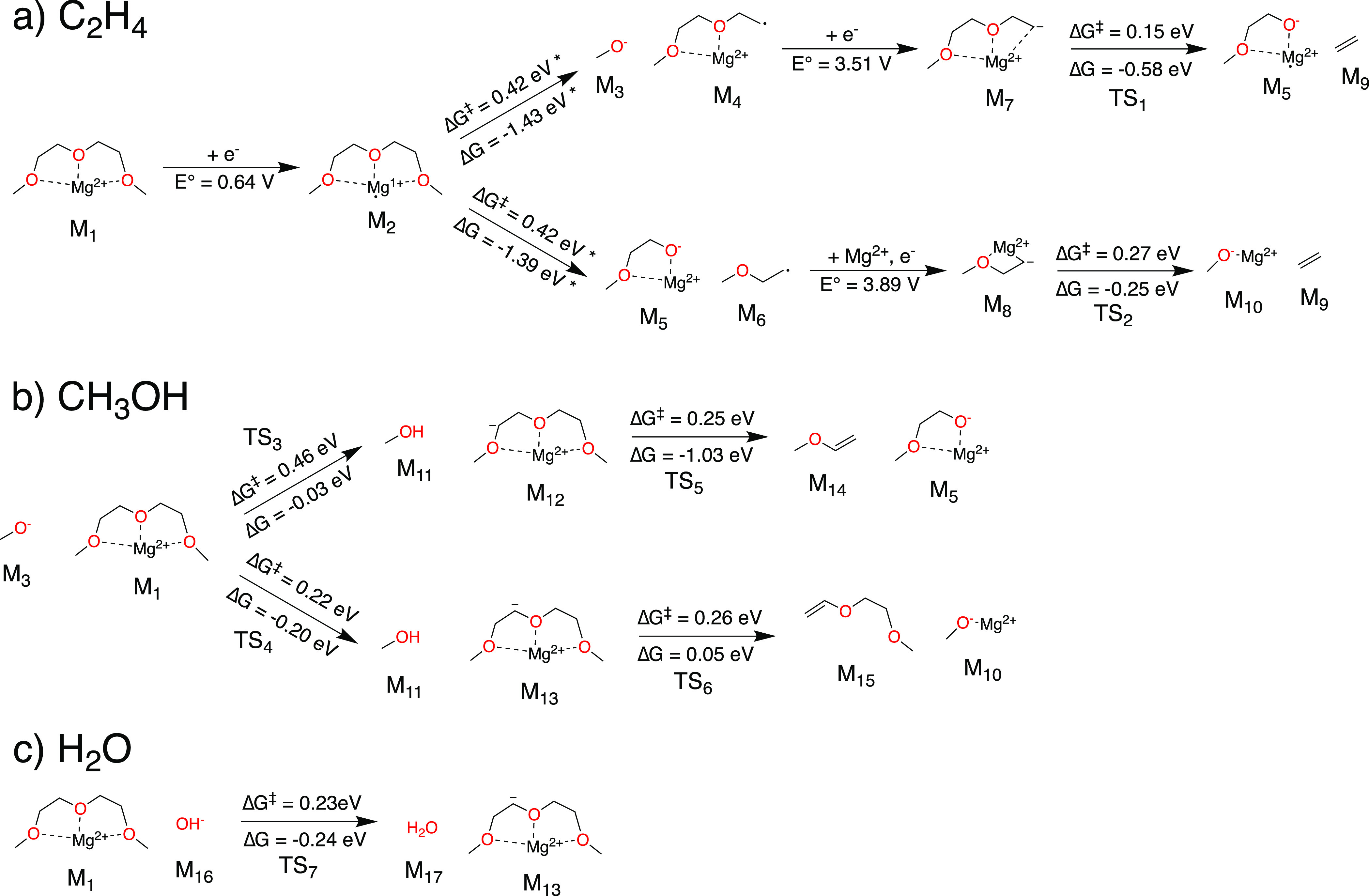
Elementary reaction mechanisms for the formation of (a) C_2_H_4_, (b) CH_3_OH, and (c) H_2_O. Reaction energies and energy barriers marked with an asterisk (*) were taken from those of Seguin et al.^27^

There are several plausible pathways that lead to the formation of C_2_H_4_ (Figure 3a). All identified pathways initialize with Mg^2+^ being partially reduced in the presence of G2 (M_1_ → M_2_). It has previously been reported that the partial reduction of Mg^2+^ ions to the highly reactive radical Mg^1+^ can promote electrolyte decomposition.^25,27^ We predict that this reduction can occur at 0.64 V vs Mg/Mg^2+^; however, this and all other reported reduction potentials with Mg ions present depend on the solvation environment of the metal ion (see the Supporting Information). Seguin et al.^27^ previously showed that the partially reduced complex M_2_ can cleave either of the internal C–O bonds with Δ*G*^⧧^ = 0.42 eV due to a bifurcation of the potential energy surface. If a methoxide ion (CH_3_O^–^, M_3_) is eliminated, we find that the remaining Mg-coordinated fragment (M_4_) can subsequently reduce (*E*° = 3.51 V) and eliminate C_2_H_4_ with a low barrier of Δ*G*^⧧^ = 0.15 eV. Alternatively, a radical CH_3_OCH_2_CH_2_^•^ (M_6_) can be eliminated. CH_3_OCH_2_CH_2_^•^ can then coordinate with an additional Mg^2+^ and reduce (M_6_ → M_8_, *E*° = 3.89 V), producing C_2_H_4_ with another low barrier (Δ*G*^⧧^ = 0.27 eV). Though this latter mechanism involving CH_3_OCH_2_CH_2_^•^ is more difficult, we nonetheless believe that it could occur, given that M_3_ + M_4_ and M_5_ + M_6_ are essentially equally likely to form from the initial cleavage of the C–O bonds in G2.

If methoxide is present, for instance, because of the mechanisms reported in Figure 3a, then the formation of methanol is facile and straightforward (Figure 3b). M_3_ can attack either methylene group in Mg-coordinated G2 (M_1_), abstracting a proton to form methanol (M_3_ + M_1_ → M_11_ + M_12_, Δ*G*^⧧^ = 0.46 eV; M_3_ + M_1_ → M_11_ + M_13_, Δ*G*^⧧^ = 0.22 eV). The deprotonated Mg-coordinated G2 species (M_12_ and M_13_) are reactive and can further decompose. M_12_ can form M_14_, methoxyethene (M_12_ → M_5_ + M_14_, Δ*G*^⧧^ = 0.25 eV). While we predict M_14_ to be a potential gaseous product (Figure 2), we do not find evidence for significant methoxyethene evolution, perhaps because the deprotonation leading to M_12_ is slower than that leading to M_13_. The decomposition of M_13_ instead produces magnesium methoxide (M_13_ → M_10_ + M_15_, Δ*G*^⧧^ = 0.26 eV), which could generate further methanol by the mechanism just described. This suggests that methanol formation in G2 electrolytes may be autocatalytic; once methoxide is initially formed, it can be continually reformed via chemical reactions with G2.

Hydroxide ions can react with Mg-coordinated G2 similarly to methoxide, abstracting a proton to form water (M_1_ + M_16_ → M_13_ + M_17_, Δ*G*^⧧^ = 0.23 eV). We note that this hydroxide could be free in the electrolyte solution (due to trace water) or could be present in the form of Mg(OH)_2_, which should be expected on Mg electrodes. Hydroxide could also potentially arise from the reduction and decomposition of CH_3_OH. The finding that G2, upon chelating Mg, can be deprotonated by hydroxide is in agreement with the prior work of Yu et al.^21^ We note that the reduction potential of water is >1.5 V vs Mg/Mg^2+^;^62^ hence, we expect that, during charging of an MIB, water should quickly reform hydroxide, creating yet another potential autocatalytic loop.

### Explaining Absent Gases

While several of the gases predicted to form via CRN analysis appear to be likely major products of G2 decomposition, namely, C_2_H_4_, CH_3_OH, and H_2_O, many of the predicted gaseous CRN products are not observed by OEMS. Just as we have used elementary reaction mechanism analysis to validate our spectroscopic peak assignment, indicating pathways that could reasonably lead to the identified gaseous CRN products, we can also suggest mechanistic explanations for why other gases are not evolved. Here, we consider three gases that were not observed experimentally in significant quantities: methane (CH_4_), ethane (C_2_H_6_), and dimethyl ether (CH_3_OCH_3_).

**Figure 4:**
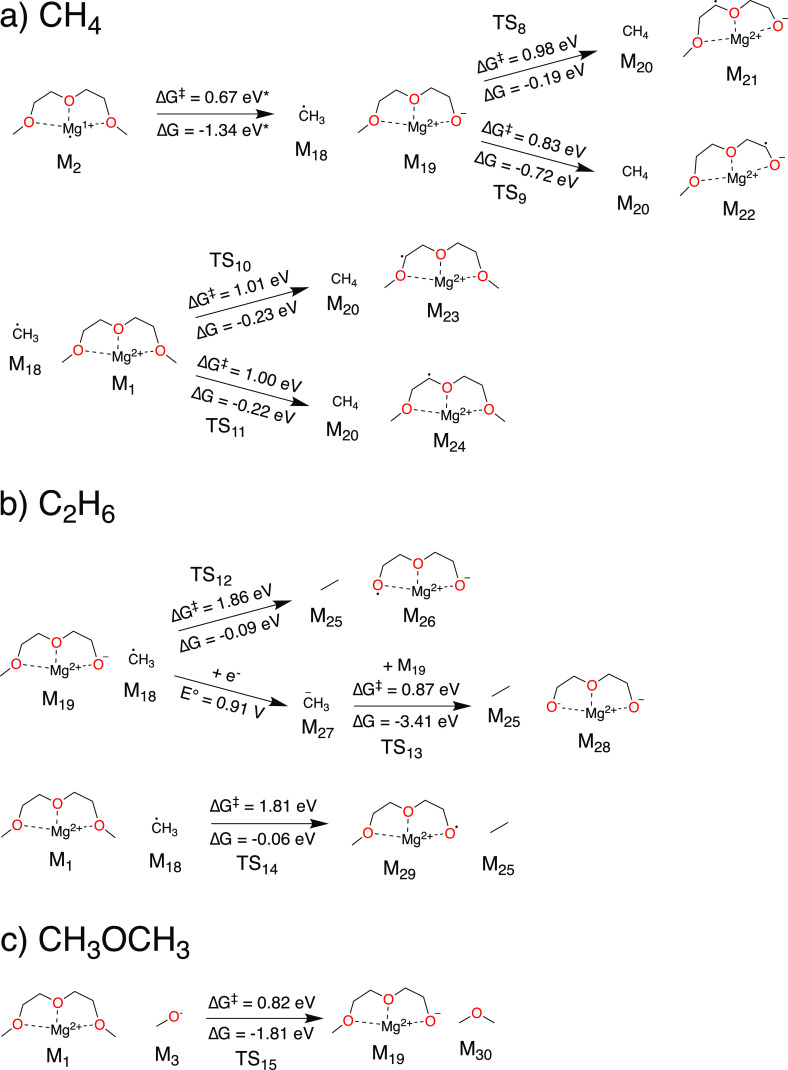
Elementary reaction mechanisms for the formation of (a) CH_4_, (b) C_2_H_6_, and (c) CH_3_OCH_3_. Reaction energies and energy barriers marked with an asterisk (*) were taken from Seguin et al.^27^

Reaction mechanisms leading to CH_4_ are shown in Figure 4a. Seguin et al. previously predicted that a methyl radical (CH_3_^•^, M_18_) could be eliminated from M_2_ with a moderate barrier (Δ*G*^⧧^ = 0.67 eV).^27^ This reaction is accessible at room temperature but is several orders of magnitude slower than the other C–O cleavage reactions discussed previously (e.g., M_2_ → M_3_ + M_4_). Even once M_18_ forms, the abstraction of H to form CH_4_ is difficult. We identified four different H abstraction reactions involving either Mg-coordinated G2 (M_1_) or a reduced and partially decomposed Mg-coordinated G2 (M_19_). The most facile abstraction (M_18_ + M_19_ → M_20_ + M_22_) has a barrier of 0.83 eV; all others have barriers of ∼1 eV.

The formation of ethane (Figure 4b) is also kinetically limited. Like CH_4_, C_2_H_6_ requires methyl radicals via the reaction M_2_ → M_18_ + M_19_. M_18_ could directly attack either M_19_ or M_1_, transferring another methyl group to form C_2_H_6_. However, these reactions suffer from extremely high barriers of ∼1.8 eV, and we therefore do not believe that they will occur under normal battery cycling conditions. If the methyl group reduces (*E*° = 0.91 V vs Mg/Mg^2+^) to form a methanide anion (M_27_, CH_3_^–^), a similar methyl transfer reaction can occur (M_27_ + M_19_ → M_25_ + M_28_); while this reaction is considerably more facile than those involving M_18_, it is still sluggish at room temperature, with Δ*G*^⧧^ = 0.87 eV. DFT is known to exhibit deficiencies in the prediction of energy barriers for radical–radical reactions, which is why we did not consider the reaction CH_3_^•^ + CH_3_^•^ → C_2_H_6_ (or M_18_ + M_18_ → M_25_). Intuitively we believe that this reaction has a low barrier or is perhaps even barrierless. However, it would require two methyl radicals to form separately in close proximity, which seems unlikely considering that the decomposition of G2 to form CH_3_^•^ is not preferred.

We find that dimethyl ether can form via methoxide (Figure 4c). The methoxide ion can attack a Mg-coordinated G2 in a single step (M_1_ + M_3_ → M_19_ + M_30_, Δ*G*^⧧^ = 0.82 eV). Because the formation of methanol by proton abstraction (e.g., M_1_ + M_3_ → M_11_ + M_13_) is considerably more facile, dimethyl ether should not be expected to form, or should form only as a minority product.

### The Role of TFSI^–^

Bistriflimide anions are known from both theoretical and experimental studies to be reductively unstable under MIB charging conditions.^22,23,25,63^ It might therefore be expected that some fragments of TFSI^–^ will be involved in gas evolution. Indeed, of the 14 potential gaseous products shown in Figure 2, four of them contain trifluoromethyl groups (−CF_3_) derived from TFSI^–^. Trifluoromethyl groups in TFSI^–^ can easily be eliminated under reducing conditions,^25^ making it reasonable to think that CF_3_ might react to form various small molecules. However, none of the major gases identified in OEMS contain −CF_3_ or any other structural motif from bistriflimide. Moreover, none of the reaction mechanisms to form C_2_H_4_, CH_3_OH, or H_2_O require TFSI^–^ or any related fragment. Although the concentration of TFSI^–^ in our OEMS experiment (1 M for a 0.5 M Mg(TFSI)_2_ electrolyte) is considerably lower than that of G2, it should be high enough for any gaseous decomposition products to be detected by OEMS.^64−66^ It therefore appears that TFSI^–^ is not significantly involved in forming any evolved gases in spite of its observed reactivity.

If bistriflimide is not forming gases or assisting in the decomposition of G2, it raises the question of what happens to the TFSI^–^ decomposition fragments. Recent AIMD results from Agarwal et al.^28^ suggest that TFSI^–^ might catastrophically decompose and even atomize at Mg interfaces, particularly if coordinated with Mg^2+^. The results of Agarwal, which are based on simulations in the presence of an idealized, completely clean Mg electrode surface (with highly undercoordinated and therefore reactive Mg), may not explain TFSI^–^ reactivity in all cases, for instance, if a robust SEI layer or even thin oxide layer is present to shield the electrolyte from a Mg metal electrode. However, in our experiment, we continuously plate Mg metal, potentially exposing fresh interfaces that can react with the electrolyte. We suggest that TFSI^–^ decomposes at this newly formed metal interface, forming primarily solid deposits rather than small molecules and gases.

**Figure 5:**
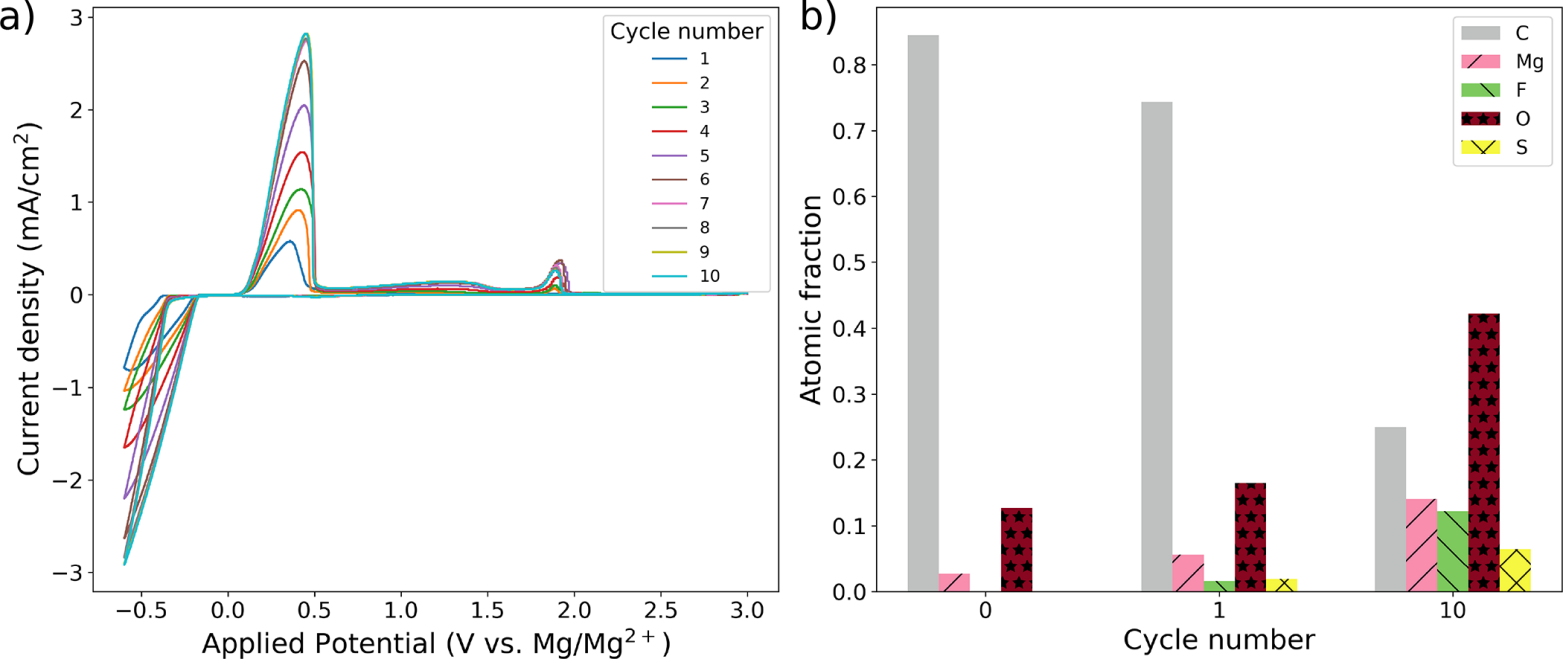
(a) Progressive cyclic voltammetry cycling behavior (10 cycles) on a fresh Pt electrode in electrochemically conditioned 0.3 M Mg(TFSI)_2_/G2 at a scan rate of 10 mV/s. (b) XPS-derived composition of the Pt electrode surface as a function of cycle number. A cycle number of 0 indicates that the measurement was taken before any potential had been applied.

Surface analysis provides further evidence that TFSI^–^ forms solid deposits on the metallic Mg surface. We cycled a 0.3 M Mg(TFSI)_2_/G2 electrolyte between −0.6 and 3.0 V vs Mg/Mg^2+^ 10 times on a Pt WE (Figure 5a) to determine if accumulation of a reaction product occurs with Mg deposition. During cycling, we used XPS to analyze the elemental composition of the surface film on the electrode (Figure 5b). Before cycling, the surface film was primarily composed of carbon (84.5%), with some oxygen (12.7%) and Mg (2.7%), and essentially no fluorine or sulfur. These results suggest that, whereas G2 (containing C, O, and H, the latter of which cannot be detected by XPS) or G2 decomposition products from conditioning might be inherently unstable at a Pt surface, TFSI^–^ (containing C, O, F, N, and S) is not inherently reactive. After the first cycle, some F (1.6%) and S (1.9%) are observed, indicating that TFSI^–^ reacts electrochemically and that the products of TFSI^–^ decomposition deposit on the electrode surface. The extent of TFSI^–^ decomposition increases upon cycling, and by the 10th cycle, the surface film is 12.2% F and 6.5% S indicating accumulation of TFSI^–^ reaction products. Notably, the atomic fraction of Mg in the surface film also increases with cycling, reflecting a degree of passivation-induced Mg stranding (Figure S17) as well as a loss of Mg inventory and battery capacity during cycling.

In addition to precipitated solid species, there is some evidence that TFSI^–^ decomposition could result in products that are soluble in G2. A recent study on the effect of impurities in MIBs with glyme solvents by Yang et al.^67^ applied electrospray ionization mass spectroscopy (ESI-MS) to study electrolyte speciation. The authors observed several F- and N-containing species in the electrolyte; because these were seen only in the conditioned electrolytes, these species could come from only TFSI^–^ decomposition.

## Conclusion

In this work, we used OEMS, CRNs, and DFT to identify gaseous byproducts of electrolyte decomposition in MIBs. From a CRN of over 6,000 species, we identified 14 possible gaseous species that could form from Mg(TFSI)_2_/G2 electrolytes. Of these, three (C_2_H_4_,CH_3_OH, and H_2_O) were consistent with major peaks in the observed OEMS spectra. We validated our peak assignments by identifying elementary reaction mechanisms for these three species, finding in all cases that the species could be easily formed via Mg-coordinated G2 (and, in the case of H_2_O, hydroxide ions). On the basis of reactive competition, we rationalized why other gases (CH_4_, C_2_H_6_, and CH_3_OCH_3_) that were predicted to form may not actually emerge during MIB cycling. Although TFSI^–^ decomposes at Mg metal electrodes and during Mg plating, we find that TFSI^–^ does not itself form any gaseous species nor is it necessary to assist in the decomposition of G2. Rather, we suggest that TFSI^–^ primarily forms solid deposits on the electrode and potentially forms some products that are soluble in G2.

The methodology described here enables facile in-depth analysis of *in situ* spectroscopy in electrochemical systems via powerful computational tools. While we have here focused on a model system in order to compare our results with previous experimental and theoretical findings, we believe that an approach mixing first-principles simulations, CRN exploration, and spectroscopy is especially well suited to allow for the characterization of completely novel electrolytes in which nothing is known regarding reactivity, decomposition products, and SEI formation. DEMS is a highly attractive point of comparison due to its high resolution, but CRN-assisted analysis of other spectroscopic measurements, such as infrared and nuclear magnetic resonance spectroscopies, should also be considered.

## Data Availability

The computational data used in this study is publicly available in a Figshare repository (DOI: 10.6084/m9.figshare.22189810.v1). Two Javascript Object Notation (JSON) files are included in this repository. madeira.json includes the properties (including structural, electronic, bonding, vibrational, and thermodynamic properties) of the 11,502 species in the MADEIRA data set. pathway_data.json includes a more limited set of structural and thermodynamic properties calculated for all minima and transition states reported in this work, labeled as they are in Figures 3 and 4.

## Supporting Information Available

Schematic of the experimental setup; cyclic voltammetry data; snapshot OEMS spectra; discussion of solvent corrections to free energy; discussion of predicted reduction potentials with and in the absence of explicit solvent; further description of CRN products; average stochastic trajectories obtained during CRN analysis; SEM images (PDF)

## References

[ref1] Ellis L. D.; Badel A. F.; Chiang M. L.; Park R. J.-Y.; Chiang Y.-M. Toward electrochemical synthesis of cement—An electrolyzer-based process for decarbonating CaCO3 while producing useful gas streams. Proc. Natl. Acad. Sci. U. S. A. 2020, 117, 12584–12591. 10.1073/pnas.1821673116. 31527245 PMC7293631

[ref2] Woldu A. R.; Huang Z.; Zhao P.; Hu L.; Astruc D. Electrochemical CO2 reduction (CO2RR) to multi-carbon products over copper-based catalysts. Coord. Chem. Rev. 2022, 454, 214340 10.1016/j.ccr.2021.214340.

[ref3] Tarpeh W. A.; Barazesh J. M.; Cath T. Y.; Nelson K. L. Electrochemical Stripping to Recover Nitrogen from Source-Separated Urine. Environ. Sci. Technol. 2018, 52, 1453–1460. 10.1021/acs.est.7b05488. 29303251

[ref4] Manzetti S.; Mariasiu F. Electric vehicle battery technologies: From present state to future systems. Renewable and Sustainable Energy Reviews 2015, 51, 1004–1012. 10.1016/j.rser.2015.07.010.

[ref5] Zhu Z.; Jiang T.; Ali M.; Meng Y.; Jin Y.; Cui Y.; Chen W. Rechargeable Batteries for Grid Scale Energy Storage. Chem. Rev. 2022, 122, 16610–16751. 10.1021/acs.chemrev.2c00289. 36150378

[ref6] Martinez Alvarado J. I.; Meinhardt J. M.; Lin S. Working at the interfaces of data science and synthetic electrochemistry. Tetrahedron Chem. 2022, 1, 100012 10.1016/j.tchem.2022.100012. 35441154 PMC9014485

[ref7] Zahrt A. F.; Mo Y.; Nandiwale K. Y.; Shprints R.; Heid E.; Jensen K. F. Machine- Learning-Guided Discovery of Electrochemical Reactions. J. Am. Chem. Soc. 2022, 144, 22599–22610. 10.1021/jacs.2c08997. 36459170 PMC9756344

[ref8] Wang L.; Menakath A.; Han F.; Wang Y.; Zavalij P. Y.; Gaskell K. J.; Borodin O.; Iuga D.; Brown S. P.; Wang C.; Xu K.; Eichhorn B. W. Identifying the components of the solid–electrolyte interphase in Li-ion batteries. Nat. Chem. 2019, 11, 789–796. 10.1038/s41557-019-0304-z. 31427766

[ref9] Lee C. W.; Cho N. H.; Im S. W.; Jee M. S.; Hwang Y. J.; Min B. K.; Nam K. T. New challenges of electrokinetic studies in investigating the reaction mechanism of electrochemical CO2 reduction. Journal of Materials Chemistry A 2018, 6, 14043–14057. 10.1039/C8TA03480J.

[ref10] Plesniak M. P.; Huang H.-M.; Procter D. J. Radical cascade reactions triggered by single electron transfer. Nature Reviews Chemistry 2017, 1, 0077 10.1038/s41570-017-0077.

[ref11] Verma P.; Maire P.; Nováak P. A review of the features and analyses of the solid electrolyte interphase in Li-ion batteries. Electrochim. Acta 2010, 55, 6332–6341. 10.1016/j.electacta.2010.05.072.

[ref12] An S. J.; Li J.; Daniel C.; Mohanty D.; Nagpure S.; Wood D. L. The state of understanding of the lithium-ion-battery graphite solid electrolyte interphase (SEI) and its relationship to formation cycling. Carbon 2016, 105, 52–76. 10.1016/j.carbon.2016.04.008.

[ref13] Olivetti E. A.; Ceder G.; Gaustad G. G.; Fu X. Lithium-Ion Battery Supply Chain Considerations: Analysis of Potential Bottlenecks in Critical Metals. Joule 2017, 1, 229–243. 10.1016/j.joule.2017.08.019.

[ref14] Sun X.; Hao H.; Hartmann P.; Liu Z.; Zhao F. Supply risks of lithium-ion battery materials: An entire supply chain estimation. Materials Today Energy 2019, 14, 100347 10.1016/j.mtener.2019.100347.

[ref15] Brown O. R.; McIntyre R. The magnesium and magnesium amalgam electrodes in aprotic organic solvents a kinetic study. Electrochim. Acta 1985, 30, 627–633. 10.1016/0013-4686(85)80104-3.

[ref16] Genders J. D.; Pletcher D. Studies using microelectrodes of the Mg(II)/Mg couple in tetrahydrofuran and propylene carbonate. Journal of Electroanalytical Chemistry and Interfacial Electrochemistry 1986, 199, 93–100. 10.1016/0022-0728(86)87044-9.

[ref17] Aurbach D.; Gofer Y.; Lu Z.; Schechter A.; Chusid O.; Gizbar H.; Cohen Y.; Ashkenazi V.; Moshkovich M.; Turgeman R.; Levi E. A short review on the comparison between Li battery systems and rechargeable magnesium battery technology. J. Power Sources 2001, 97–98, 28–32. 10.1016/S0378-7753(01)00585-7.

[ref18] Shterenberg I.; Salama M.; Yoo H. D.; Gofer Y.; Park J.-B.; Sun Y.-K.; Aurbach D. Evaluation of (CF3SO2)2N (TFSI) Based Electrolyte Solutions for Mg Batteries. J. Electrochem. Soc. 2015, 162, A7118 10.1149/2.0161513jes.

[ref19] Arthur T. S.; Glans P.-A.; Singh N.; Tutusaus O.; Nie K.; Liu Y.-S.; Mizuno F.; Guo J.; Alsem D. H.; Salmon N. J.; Mohtadi R. Interfacial Insight from Operando XAS/TEM for Magnesium Metal Deposition with Borohydride Electrolytes. Chem. Mater. 2017, 29, 7183–7188. 10.1021/acs.chemmater.7b01189.

[ref20] Gao T.; Hou S.; Huynh K.; Wang F.; Eidson N.; Fan X.; Han F.; Luo C.; Mao M.; Li X.; Wang C. Existence of Solid Electrolyte Interphase in Mg Batteries: Mg/S Chemistry as an Example. ACS Appl. Mater. Interfaces 2018, 10, 14767–14776. 10.1021/acsami.8b02425. 29620854

[ref21] Yu Y.; Baskin A.; Valero-Vidal C.; Hahn N. T.; Liu Q.; Zavadil K. R.; Eichhorn B. W.; Prendergast D.; Crumlin E. J. Instability at the Electrode/Electrolyte Interface Induced by Hard Cation Chelation and Nucleophilic Attack. Chem. Mater. 2017, 29, 8504–8512. 10.1021/acs.chemmater.7b03404.

[ref22] Yoo H. D.; Han S.-D.; Bolotin I. L.; Nolis G. M.; Bayliss R. D.; Burrell A. K.; Vaughey J. T.; Cabana J. Degradation Mechanisms of Magnesium Metal Anodes in Electrolytes Based on (CF3SO2)2N– at High Current Densities. Langmuir 2017, 33, 9398–9406. 10.1021/acs.langmuir.7b01051. 28636826

[ref23] Jay R.; Tomich A. W.; Zhang J.; Zhao Y.; De Gorostiza A.; Lavallo V.; Guo J. Comparative Study of Mg(CB11H12)2 and Mg(TFSI)2 at the Magnesium/Electrolyte Interface. ACS Appl. Mater. Interfaces 2019, 11, 11414–11420. 10.1021/acsami.9b00037. 30860349

[ref24] Nguyen D.-T.; Eng A. Y. S.; Ng M.-F.; Kumar V.; Sofer Z.; Handoko A. D.; Subramanian G. S.; Seh Z. W. A High-Performance Magnesium Triflate-based Electrolyte for Rechargeable Magnesium Batteries. Cell Reports Physical Science 2020, 1, 100265 10.1016/j.xcrp.2020.100265.

[ref25] Rajput N. N.; Qu X.; Sa N.; Burrell A. K.; Persson K. A. The Coupling between Stability and Ion Pair Formation in Magnesium Electrolytes from First-Principles Quantum Mechanics and Classical Molecular Dynamics. J. Am. Chem. Soc. 2015, 137, 3411–3420. 10.1021/jacs.5b01004. 25668289

[ref26] Lowe J. S.; Siegel D. J. Reaction Pathways for Solvent Decomposition on Magnesium Anodes. J. Phys. Chem. C 2018, 122, 10714–10724. 10.1021/acs.jpcc.8b01752.

[ref27] Seguin T. J.; Hahn N. T.; Zavadil K. R.; Persson K. A. Elucidating Non-aqueous Solvent Stability and Associated Decomposition Mechanisms for Mg Energy Storage Applications From First-Principles. Frontiers in Chemistry 2019, 7, 175 10.3389/fchem.2019.00175. 31024883 PMC6465547

[ref28] Agarwal G.; Howard J. D.; Prabhakaran V.; Johnson G. E.; Murugesan V.; Mueller K. T.; Curtiss L. A.; Assary R. S. Insights into Spontaneous Solid Electrolyte Interphase Formation at Magnesium Metal Anode Surface from Ab Initio Molecular Dynamics Simulations. ACS Appl. Mater. Interfaces 2021, 13, 38816–38825. 10.1021/acsami.1c07864. 34362250

[ref29] Novák P.; Panitz J. C.; Joho F.; Lanz M.; Imhof R.; Coluccia M. Advanced in situ methods for the characterization of practical electrodes in lithium-ion batteries. J. Power Sources 2000, 90, 52–58. 10.1016/S0378-7753(00)00447-X.

[ref30] McCloskey B. D.; Bethune D. S.; Shelby R. M.; Girishkumar G.; Luntz A. C. Solvents’ Critical Role in Nonaqueous Lithium–Oxygen Battery Electrochemistry. J. Phys. Chem. Lett. 2011, 2, 1161–1166. 10.1021/jz200352v. 26295320

[ref31] Tsiouvaras N.; Meini S.; Buchberger I.; Gasteiger H. A. A Novel On-Line Mass Spectrometer Design for the Study of Multiple Charging Cycles of a Li-O2 Battery. J. Electrochem. Soc. 2013, 160, A471 10.1149/2.042303jes.

[ref32] Nie K.; Wang X.; Qiu J.; Wang Y.; Yang Q.; Xu J.; Yu X.; Li H.; Huang X.; Chen L. Increasing Poly(ethylene oxide) Stability to 4.5 V by Surface Coating of the Cathode. ACS Energy Letters 2020, 5, 826–832. 10.1021/acsenergylett.9b02739.

[ref33] Shi B.; Liu K.; Lee E.; Liao C. Batteries: Materials principles and characterization methods; IOP Publishing: 2021; Chapter 5: Differential electrochemical mass spectrometry (DEMS) for batteries.

[ref34] Wen M.; Spotte-Smith E. W. C.; Blau S. M.; McDermott M. J.; Krishnapriyan A. S.; Persson K. A. Chemical reaction networks and opportunities for machine learning. Nature Computational Science 2023, 3, 12–24. 10.1038/s43588-022-00369-z. 38177958

[ref35] Barter D.; Spotte-Smith E. W. C.; Redkar N. S.; Khanwale A.; Dwaraknath S.; Persson K. A.; Blau S. M. Predictive stochastic analysis of massive filter-based electrochemical reaction networks. Digital Discovery 2023, 2, 123–137. 10.1039/D2DD00117A.

[ref36] Spotte-Smith E. W. C.; Blau S. M.; Xie X.; Patel H. D.; Wen M.; Wood B.; Dwaraknath S.; Persson K. A. Quantum chemical calculations of lithium-ion battery electrolyte and interphase species. Scientific Data 2021, 8, 203 10.1038/s41597-021-00986-9. 34354089 PMC8342431

[ref37] Mardirossian N.; Head-Gordon M. B97X-V: A 10-parameter, range-separated hybrid, generalized gradient approximation density functional with nonlocal correlation, designed by a survival-of-the-fittest strategy. Phys. Chem. Chem. Phys. 2014, 16, 9904–9924. 10.1039/c3cp54374a. 24430168

[ref38] Rappoport D.; Furche F. Property-optimized Gaussian basis sets for molecular response calculations. J. Chem. Phys. 2010, 133, 134105 10.1063/1.3484283. 20942521

[ref39] Marenich A. V.; Cramer C. J.; Truhlar D. G. Universal Solvation Model Based on Solute Electron Density and on a Continuum Model of the Solvent Defined by the Bulk Dielectric Constant and Atomic Surface Tensions. J. Phys. Chem. B 2009, 113, 6378–6396. 10.1021/jp810292n. 19366259

[ref40] Qu X.; Jain A.; Rajput N. N.; Cheng L.; Zhang Y.; Ong S. P.; Brafman M.; Maginn E.; Curtiss L. A.; Persson K. A. The Electrolyte Genome project: A big data approach in battery materials discovery. Comput. Mater. Sci. 2015, 103, 56–67. 10.1016/j.commatsci.2015.02.050.

[ref41] Epifanovsky E.; Gilbert A. T. B.; Feng X.; Lee J.; Mao Y.; Mardirossian N.; Pokhilko P.; White A. F.; Coons M. P.; Dempwolff A. L.; Gan Z.; Hait D.; Horn P. R.; Jacobson L. D.; Kaliman I.; Kussmann J.; Lange A. W.; Lao K. U.; Levine D. S.; Liu J.; McKenzie S. C.; Morrison A. F.; Nanda K. D.; Plasser F.; Rehn D. R.; Vidal M. L.; You Z.-Q.; Zhu Y.; Alam B.; Albrecht B. J.; Aldossary A.; Alguire E.; Andersen J. H.; Athavale V.; Barton D.; Begam K.; Behn A.; Bellonzi N.; Bernard Y. A.; Berquist E. J.; Burton H. G. A.; Carreras A.; Carter-Fenk K.; Chakraborty R.; Chien A. D.; Closser K. D.; Cofer-Shabica V.; Dasgupta S.; de Wergifosse M.; Deng J.; Diedenhofen M.; Do H.; Ehlert S.; Fang P.-T.; Fatehi S.; Feng Q.; Friedhoff T.; Gayvert J.; Ge Q.; Gidofalvi G.; Goldey M.; Gomes J.; Gonz’alez-Espinoza C. E.; Gulania S.; Gunina A. O.; Hanson-Heine M. W. D.; Harbach P. H. P.; Hauser A.; Herbst M. F.; Hernández Vera M.; Hodecker M.; Holden Z. C.; Houck S.; Huang X.; Hui K.; Huynh B. C.; Ivanov M.; Jász; Ji H.; Jiang H.; Kaduk B.; Kähler S.; Khistyaev K.; Kim J.; Kis G.; Klunzinger P.; Koczor-Benda Z.; Koh J. H.; Kosenkov D.; Koulias L.; Kowalczyk T.; Krauter C. M.; Kue K.; Kunitsa A.; Kus T.; Ladjánszki I.; Landau A.; Lawler K. V.; Lefrancois D.; Lehtola S.; Li R. R.; Li Y.-P.; Liang J.; Liebenthal M.; Lin H.-H.; Lin Y.-S.; Liu F.; Liu K.-Y.; Loipersberger M.; Luenser A.; Manjanath A.; Manohar P.; Mansoor E.; Manzer S. F.; Mao S.-P.; Marenich A. V.; Markovich T.; Mason S.; Maurer S. A.; McLaughlin P. F.; Menger M. F. S. J.; Mewes J.-M.; Mewes S. A.; Morgante P.; Mullinax J. W.; Oosterbaan K. J.; Paran G.; Paul A. C.; Paul S. K.; Pavošević F.; Pei Z.; Prager S.; Proynov E. I.; Rák; Ramos-Cordoba E.; Rana B.; Rask A. E.; Rettig A.; Richard R. M.; Rob F.; Rossomme E.; Scheele T.; Scheurer M.; Schneider M.; Sergueev N.; Sharada S. M.; Skomorowski W.; Small D. W.; Stein C. J.; Su Y.-C.; Sundstrom E. J.; Tao Z.; Thirman J.; Tornai G. J.; Tsuchimochi T.; Tubman N. M.; Veccham S. P.; Vydrov O.; Wenzel J.; Witte J.; Yamada A.; Yao K.; Yeganeh S.; Yost S. R.; Zech A.; Zhang I. Y.; Zhang X.; Zhang Y.; Zuev D.; Aspuru-Guzik A.; Bell A. T.; Besley N. A.; Bravaya K. B.; Brooks B. R.; Casanova D.; Chai J.-D.; Coriani S.; Cramer C. J.; Cserey G.; DePrince A. E.; DiStasio R. A.; Dreuw A.; Dunietz B. D.; Furlani T. R.; Goddard W. A.; Hammes-Schiffer S.; Head-Gordon T.; Hehre W. J.; Hsu C.-P.; Jagau T.-C.; Jung Y.; Klamt A.; Kong J.; Lambrecht D. S.; Liang W.; Mayhall N. J.; McCurdy C. W.; Neaton J. B.; Ochsenfeld C.; Parkhill J. A.; Peverati R.; Rassolov V. A.; Shao Y.; Slipchenko L. V.; Stauch T.; Steele R. P.; Subotnik J. E.; Thom A. J. W.; Tkatchenko A.; Truhlar D. G.; Van Voorhis T.; Wesolowski T. A.; Whaley K. B.; Woodcock H. L.; Zimmerman P. M.; Faraji S.; Gill P. M. W.; Head-Gordon M.; Herbert J. M.; Krylov A. I. Software for the frontiers of quantum chemistry: An overview of developments in the Q-Chem 5 package. J. Chem. Phys. 2021, 155, 084801 10.1063/5.0055522. 34470363 PMC9984241

[ref42] Mathew K.; Montoya J. H.; Faghaninia A.; Dwarakanath S.; Aykol M.; Tang H.; Chu I.-h.; Smidt T.; Bocklund B.; Horton M.; Dagdelen J.; Wood B.; Liu Z.-K.; Neaton J.; Ong S. P.; Persson K.; Jain A. Atomate: A high-level interface to generate, execute, and analyze computational materials science workflows. Comput. Mater. Sci. 2017, 139, 140–152. 10.1016/j.commatsci.2017.07.030.

[ref43] Ong S. P.; Richards W. D.; Jain A.; Hautier G.; Kocher M.; Cholia S.; Gunter D.; Chevrier V. L.; Persson K. A.; Ceder G. Python Materials Genomics (pymatgen): A robust, open-source python library for materials analysis. Comput. Mater. Sci. 2013, 68, 314–319. 10.1016/j.commatsci.2012.10.028.

[ref44] Blau S.; Spotte-Smith E. W. C.; Wood B.; Dwaraknath S.; Persson K. Accurate, Automated Density Functional Theory for Complex Molecules Using On-the-fly Error Correction ChemRxiv, 2020. 10.26434/chemrxiv.13076030.v1 (accessed April 10, 2023).

[ref45] Spotte-Smith E. W. C.; Blau S. M.; Barter D.; Leon N. J.; Hahn N. T.; Redkar N. S.; Zavadil K. R.; Liao C.; Persson K. A. Data for “Chemical Reaction Networks Explain Gas Evolution Mechanisms in Mg-Ion Batteries”. Figshare, Data set, 2023. 10.6084/m9.figshare.22189810.v1 (accessed April 10, 2023). PMC10251523 37235548

[ref46] Chai J.-D.; Head-Gordon M. Long-range corrected hybrid density functionals with damped atom–atom dispersion corrections. Phys. Chem. Chem. Phys. 2008, 10, 6615–6620. 10.1039/b810189b. 18989472

[ref47] Klamt A. The COSMO and COSMO-RS solvation models. WIREs Computational Molecular Science 2011, 1, 699–709. 10.1002/wcms.56.

[ref48] Weinhold F.; Glendening E. D. NBO 5.0 program manual: natural bond orbital analysis programs; Theoretical Chemistry Institute and Department of Chemistry, University of Wisconsin: Madison, WI, 2001.

[ref49] Shang C.; Liu Z.-P. Stochastic Surface Walking Method for Structure Prediction and Pathway Searching. J. Chem. Theory Comput. 2013, 9, 1838–1845. 10.1021/ct301010b. 26587640

[ref50] Spotte-Smith E. W. C.; Kam R. L.; Barter D.; Xie X.; Hou T.; Dwaraknath S.; Blau S. M.; Persson K. A. Toward a Mechanistic Model of Solid–Electrolyte Interphase Formation and Evolution in Lithium-Ion Batteries. ACS Energy Letters 2022, 7, 1446–1453. 10.1021/acsenergylett.2c00517.

[ref51] Gillespie D. T. Exact stochastic simulation of coupled chemical reactions. J. Phys. Chem. 1977, 81, 2340–2361. 10.1021/j100540a008.

[ref52] Gillespie D. T. Stochastic Simulation of Chemical Kinetics. Annu. Rev. Phys. Chem. 2007, 58, 35–55. 10.1146/annurev.physchem.58.032806.104637. 17037977

[ref53] Jacobson L. D.; Bochevarov A. D.; Watson M. A.; Hughes T. F.; Rinaldo D.; Ehrlich S.; Steinbrecher T. B.; Vaitheeswaran S.; Philipp D. M.; Halls M. D.; Friesner R. A. Automated Transition State Search and Its Application to Diverse Types of Organic Reactions. J. Chem. Theory Comput. 2017, 13, 5780–5797. 10.1021/acs.jctc.7b00764. 28957627

[ref54] Bochevarov A. D.; Harder E.; Hughes T. F.; Greenwood J. R.; Braden D. A.; Philipp D. M.; Rinaldo D.; Halls M. D.; Zhang J.; Friesner R. A. Jaguar: A highperformance quantum chemistry software program with strengths in life and materials sciences. Int. J. Quantum Chem. 2013, 113, 2110–2142. 10.1002/qua.24481.

[ref55] Mardirossian N.; Head-Gordon M. B97M-V: A combinatorially optimized, rangeseparated hybrid, meta-GGA density functional with VV10 nonlocal correlation. J. Chem. Phys. 2016, 144, 214110 10.1063/1.4952647. 27276948

[ref56] Mardirossian N.; Head-Gordon M. Thirty years of density functional theory in computational chemistry: an overview and extensive assessment of 200 density functionals. Mol. Phys. 2017, 115, 2315–2372. 10.1080/00268976.2017.1333644.

[ref57] Pankow J. F.; Asher W. E. SIMPOL.1: a simple group contribution method for predicting vapor pressures and enthalpies of vaporization of multifunctional organic compounds. Atmospheric Chemistry and Physics 2008, 8, 2773–2796. 10.5194/acp-8-2773-2008.

[ref58] Topping D.; Barley M.; Bane M. K.; Higham N.; Aumont B.; Dingle N.; McFiggans G. UManSysProp v1.0: an online and open-source facility for molecular property prediction and atmospheric aerosol calculations. Geoscientific Model Development 2016, 9, 899–914. 10.5194/gmd-9-899-2016.

[ref59] Bachhav M. N.; Hahn N. T.; Zavadil K. R.; Nelson E. G.; Crowe A. J.; Bartlett B. M.; Chu P.-W.; Araullo-Peters V. J.; Marquis E. A. Microstructure and chemistry of electrodeposited Mg films. J. Electrochem. Soc. 2016, 163, D645 10.1149/2.0181613jes.

[ref60] Connell J. G.; Genorio B.; Lopes P. P.; Strmcnik D.; Stamenkovic V. R.; Markovic N. M. Tuning the Reversibility of Mg Anodes via Controlled Surface Passivation by H2O/Cl– in Organic Electrolytes. Chem. Mater. 2016, 28, 8268–8277. 10.1021/acs.chemmater.6b03227.

[ref61] Coplen T. B.; Böhlke J. K.; De Bievre P.; Ding T.; Holden N.; Hopple J.; Krouse H.; Lamberty A.; Peiser H.; Revesz K.; et al. Isotope-abundance variations of selected elements (IUPAC Technical Report). Pure and applied chemistry 2002, 74, 1987–2017. 10.1351/pac200274101987.

[ref62] Haynes W.; Lide D.; Bruno T. CRC handbook of chemistry and physics; CRC Press: 2012.

[ref63] Baskin A.; Prendergast D. Exploration of the Detailed Conditions for Reductive Stability of Mg(TFSI)2 in Diglyme: Implications for Multivalent Electrolytes. J. Phys. Chem. C 2016, 120, 3583–3594. 10.1021/acs.jpcc.5b08999.

[ref64] Michalak B.; Berkes B. B.; Sommer H.; Bergfeldt T.; Brezesinski T.; Janek J. Gas evolution in LiNi0. 5Mn1. 5O4/graphite cells studied in operando by a combination of differential electrochemical mass spectrometry, neutron imaging, and pressure measurements. Analytical chemistry 2016, 88, 2877–2883. 10.1021/acs.analchem.5b04696. 26813026

[ref65] Pritzl D.; Solchenbach S.; Wetjen M.; Gasteiger H. A. Analysis of vinylene carbonate (VC) as additive in graphite/LiNi0. 5Mn1. 5O4 cells. J. Electrochem. Soc. 2017, 164, A2625 10.1149/2.1441712jes.

[ref66] Xu G.; Wang X.; Li J.; Shangguan X.; Huang S.; Lu D.; Chen B.; Ma J.; Dong S.; Zhou X.; et al. Tracing the impact of hybrid functional additives on a high-voltage (5 V-class) SiO x-C/LiNi0. 5Mn1. 5O4 Li-ion battery system. Chem. Mater. 2018, 30, 8291–8302. 10.1021/acs.chemmater.8b03764.

[ref67] Yang Z.; Yang M.; Hahn N. T.; Connell J.; Bloom I.; Liao C.; Ingram B. J.; Trahey L. Toward practical issues: Identification and mitigation of the impurity effect in glyme solvents on the reversibility of Mg plating/stripping in Mg batteries. Frontiers in Chemistry 2022, 10, 966332 10.3389/fchem.2022.966332. 36034674 PMC9413053

