# Supplementary material for: Chemical Reaction Networks Explain Gas Evolution Mechanisms in Mg-Ion Batteries
Source: J Am Chem Soc. 2023 May 26;145(22):12181–92. doi: 10.1021/jacs.3c02222 (PMC10251523; doi:10.1021/jacs.3c02222)
Supplement: Supplementary file 1 — ja3c02222_si_001.pdf [file ja3c02222_si_001.pdf]

Supporting Information: Chemical Reaction Networks Explain Gas Evolution Mechanisms in Mg-Ion Batteries

Evan Walter Clark Spotte-Smith,[†,‡] Samuel M. Blau,[¶] Daniel Barter,[¶] Noel J. Leon,[§] Nathan T. Hahn,[‖] Nikita S. Redkar,[⊥] Kevin R. Zavadil,[‖] Chen Liao,[*,§] and Kristin A. Persson[*,‡,#]

†*Materials Science Division, Lawrence Berkeley National Laboratory, 1 Cyclotron Road, Berkeley, CA, 94720 USA*

‡*Department of Materials Science and Engineering, University of California, Berkeley, 210 Hearst Memorial Mining Building, Berkeley, CA, 94720 USA*

¶*Energy Storage and Distributed Resources, Lawrence Berkeley National Laboratory, 1 Cyclotron Road, Berkeley, CA, 94720 USA*

§*Argonne National Laboratory, 9700 S. Cass Ave, Lemont, IL 60439 USA*

‖*Material, Physical and Chemical Sciences Center, Sandia National Laboratories, 1515 Eubank Blvd. SE, Albuquerque, NM 87123 USA*

⊥*Department of Chemical and Biomolecular Engineering, University of California, Berkeley, 201 Gilman Hall, Berkeley, CA, 94720 USA*

#*Molecular Foundry, Lawrence Berkeley National Laboratory, 1 Cyclotron Road, Berkeley, CA, 94720 USA*

E-mail: liaoc@anl.gov; kapersson@lbl.gov

Experimental Schematic

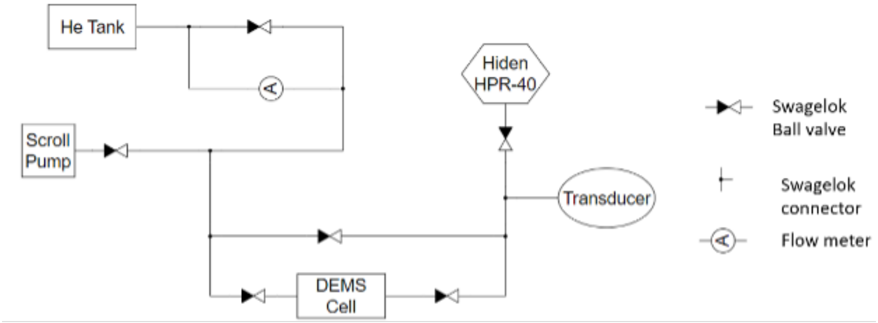

Figure S1: Schematic drawing of the OEMS system used in this study.

Demonstration of Reversible Mg Plating and Stripping

Figure S2 shows data from a cyclic voltammetry (CV) experiment conducted on the same MIB cell used for OEMS measurements. A scan of 100 mV s^{-1} was applied to the cell with Au WE, Mg RE, and a 0.5 M solution of $Mg(TFSI)_2$ dissolved in G2. Four cyclces are shown. All cycles demonstrate reversible Mg plating and stripping. This confirms that, during the OEMS experiment in which the cell voltage was held at -1.0 V, Mg was constantly plated.

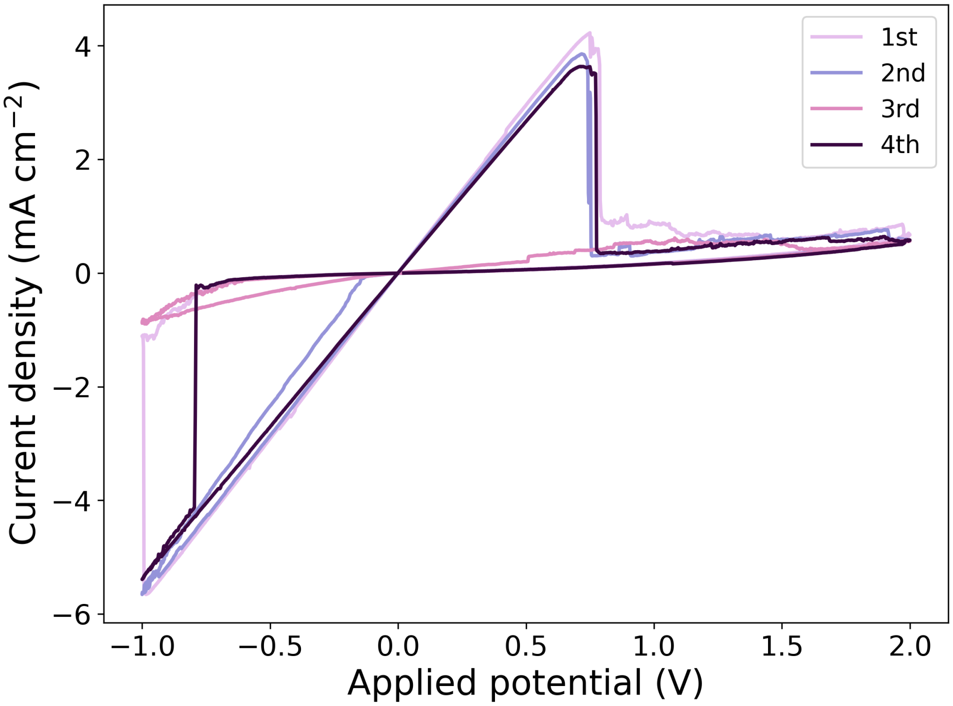

Figure S2: Cyclic voltammogram showing two plating-stripping cycles for a DEMS cell containing a 0.5M $Mg(TFSI)_2$/G2 electrolyte, an Au working electrode, and Mg foil reference electrode. The scan rate was 100 mV s^{-1}.

Snapshot OEMS Spectra

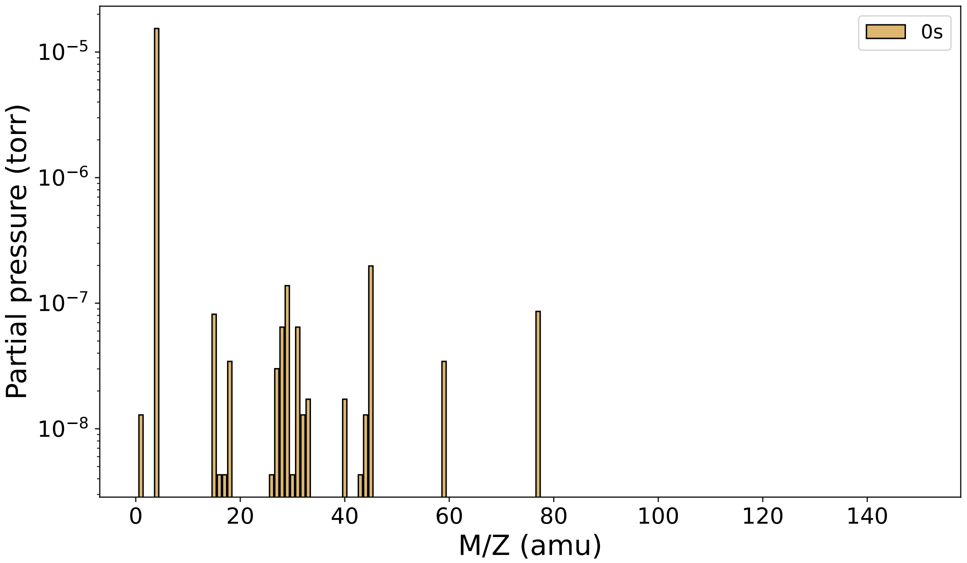

Figure S3: Snapshot OEMS spectrum taken at the beginning of a potentiostatic experiment.

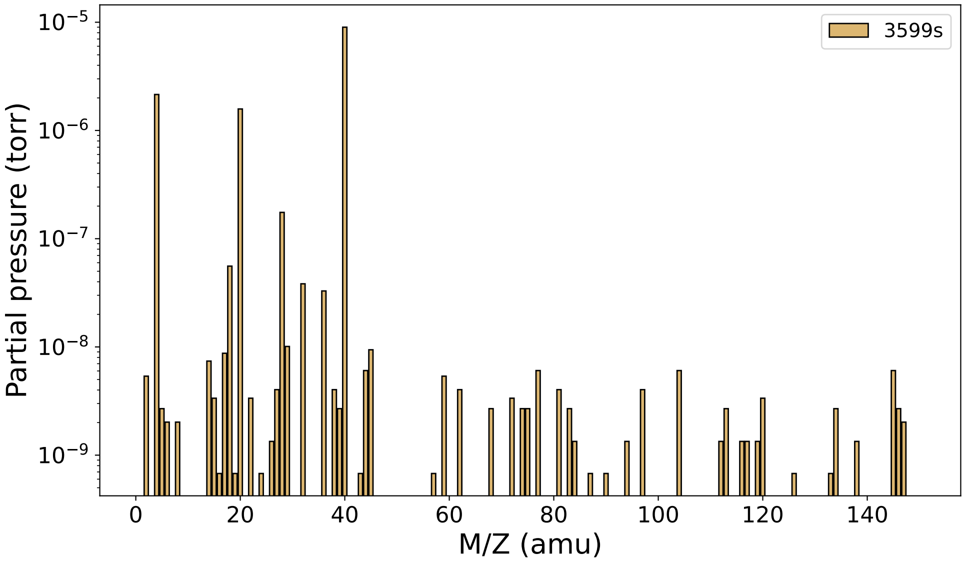

Figure S4: Snapshot OEMS spectrum taken one hour into a potentiostatic experiment.

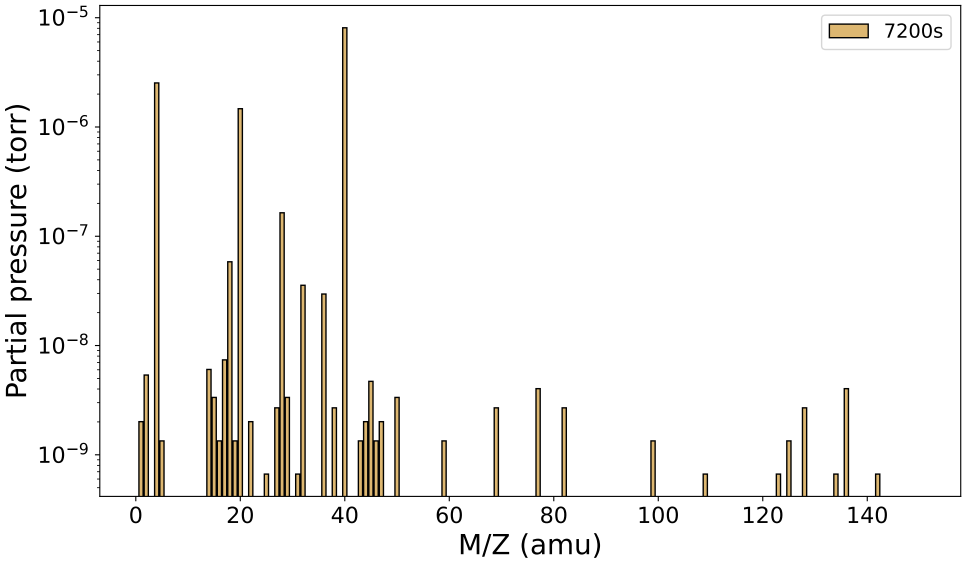

Figure S5: Snapshot OEMS spectrum taken two hours into a potentiostatic experiment.

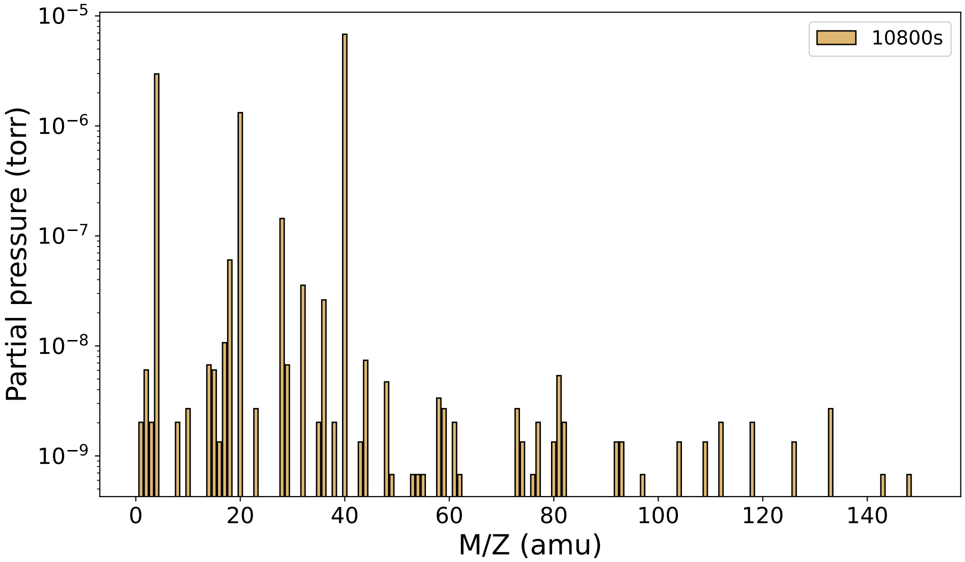

Figure S6: Snapshot OEMS spectrum taken three hours into a potentiostatic experiment.

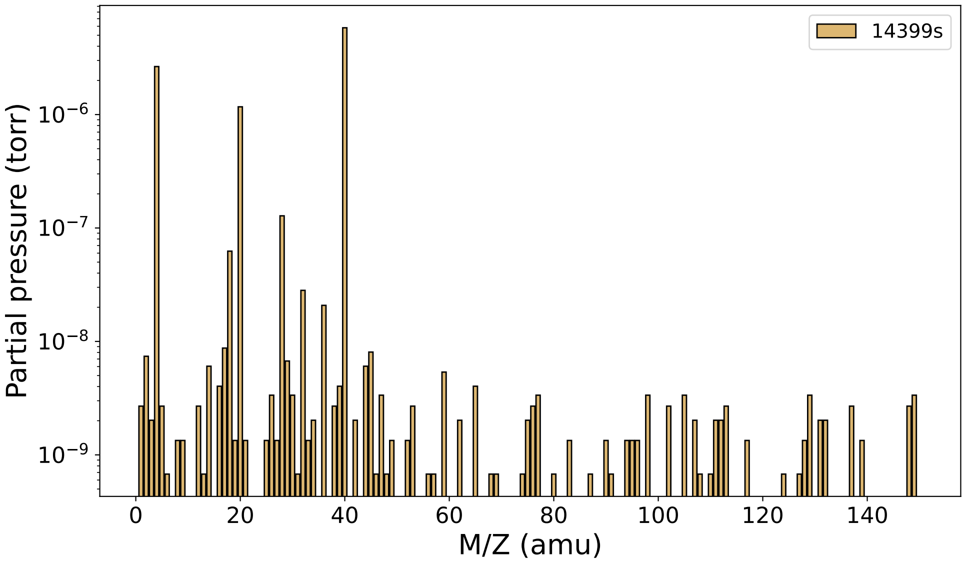

Figure S7: Snapshot OEMS spectrum taken four hours into a potentiostatic experiment.

Average Monte Carlo Trajectories

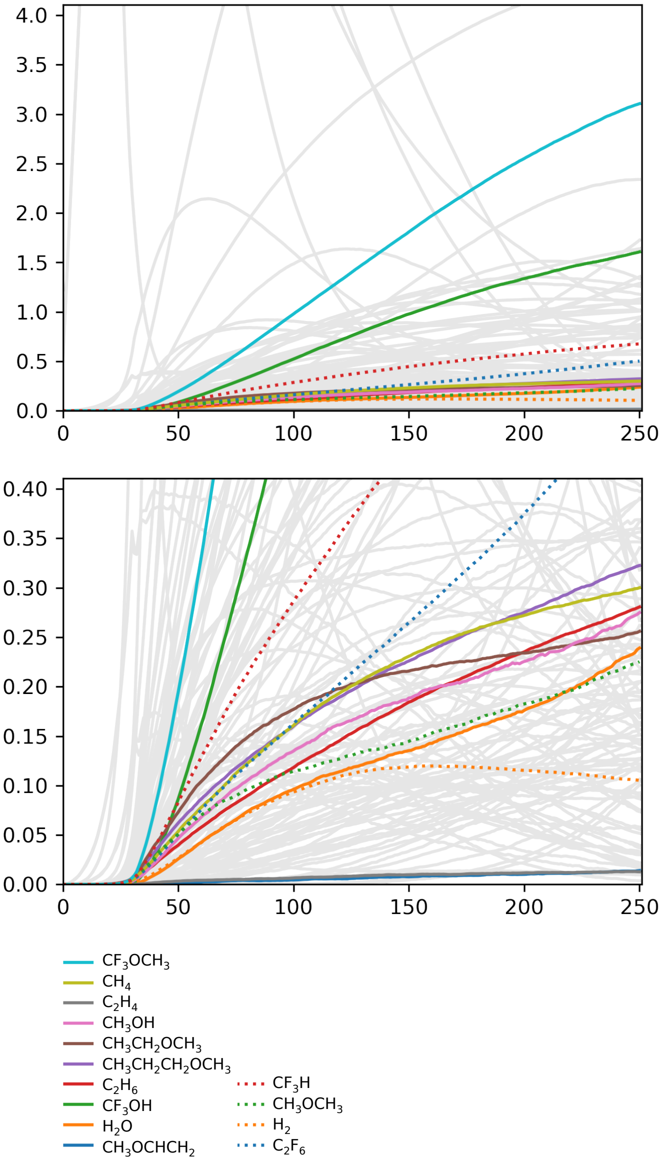

Figure S8: Average of 50,000 Monte Carlo trajectories beginning with Mg^{2+}, G2, and $TFSI^-$ subjected to a potential of 0V vs. Mg/Mg^{2+}. Predicted gaseous products are highlighted.

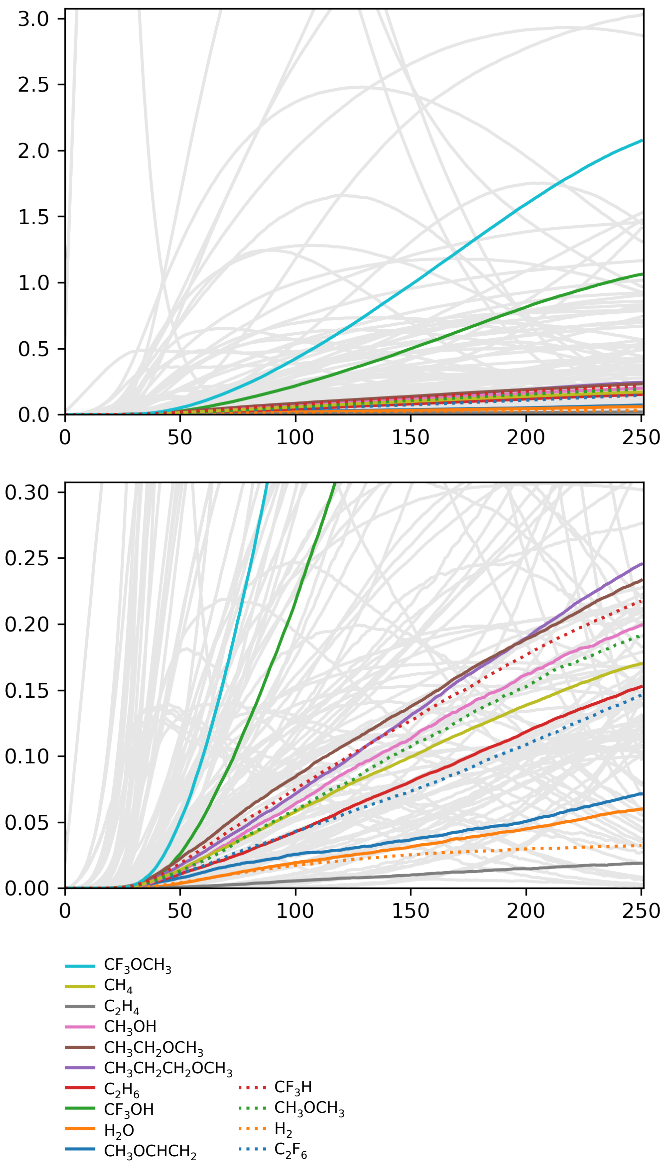

Figure S9: Average of 50,000 Monte Carlo trajectories beginning with Mg^{2+}, G2, $TFSI^-$, and CO_2 subjected to a potential of 0V vs. Mg/Mg^{2+}. Predicted gaseous products are highlighted.

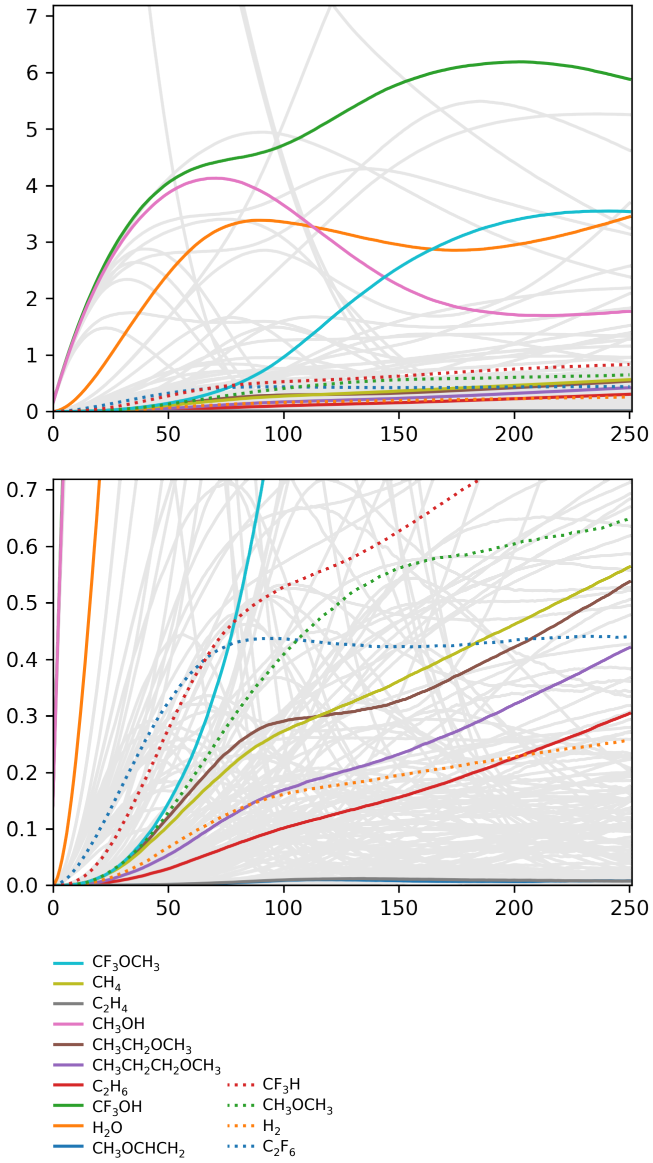

Figure S10: Average of 50,000 Monte Carlo trajectories beginning with Mg^{2+}, G2, $TFSI^{-}$, and OH^{-} subjected to a potential of 0V vs. Mg/Mg^{2+}. Predicted gaseous products are highlighted.

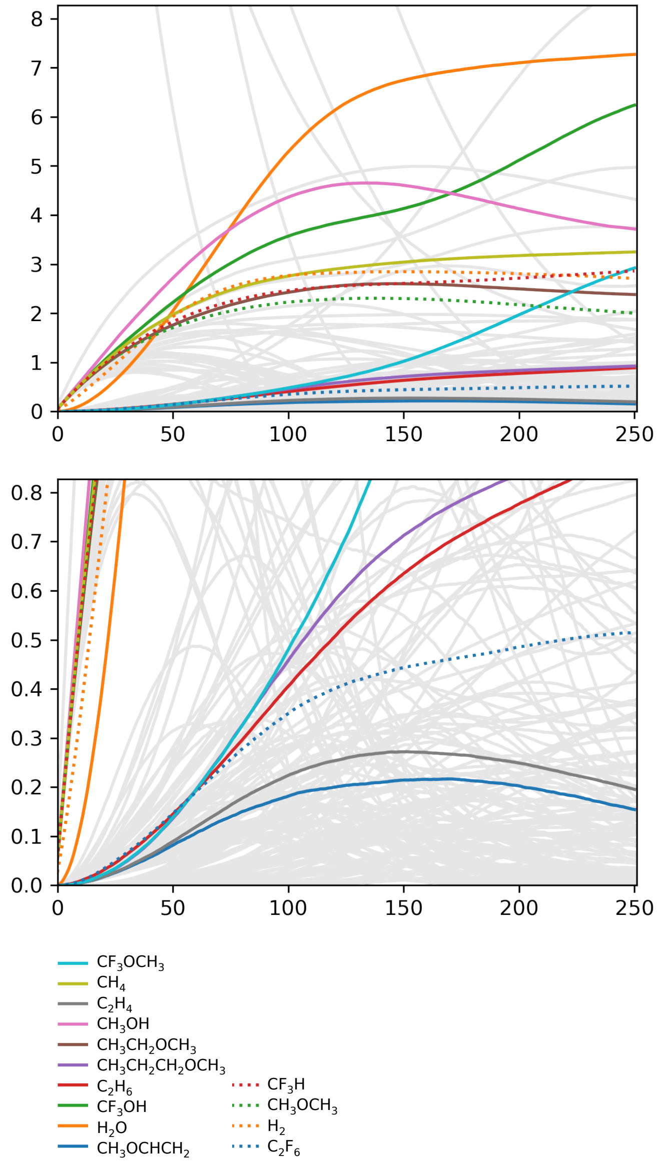

Figure S11: Average of 50,000 Monte Carlo trajectories beginning with Mg^{2+}, G2, $TFSI^{-}$, OH^{-}, and $H^{\bullet}$ subjected to a potential of 0V vs. Mg/Mg^{2+}. Predicted gaseous products are highlighted.

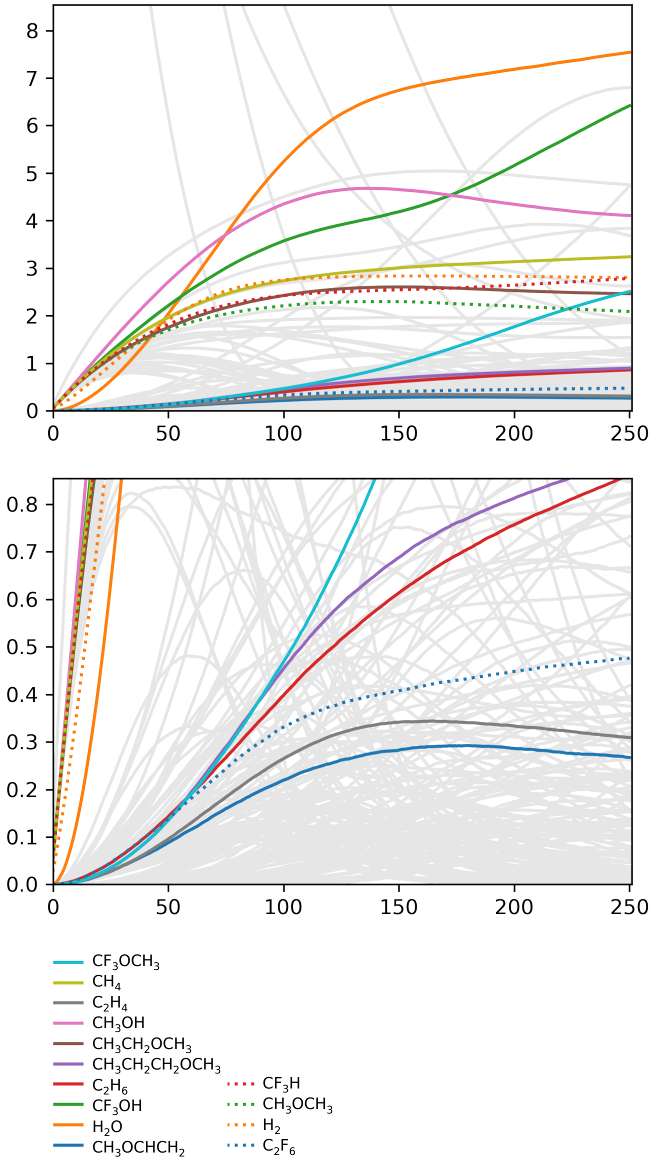

Figure S12: Average of 50,000 Monte Carlo trajectories beginning with Mg^{2+}, G2, $TFSI^-$, CO_2, OH^-, and $H^\bullet$ subjected to a potential of 0V vs. Mg/Mg^{2+}. Predicted gaseous products are highlighted.

Solvation Correction

We optimized clusters with variable numbers of G2 as well as dimethoxyethane (DME) coordinating Mg^{2+} and Mg^{1+} ions in Q-Chem at the ωB97X-D/def2-SVPD/PCM//ωB97X-V/def2-TZVPPD/SMD level of theory.[1–5] From these clusters, we can calculate the relative stabilization of the ion as

$$\Delta E_{stabilization} = E_{S_n} - E_{S_0} - nE_S \tag{1}$$

where E_{S_n} is the electronic energy of the ion surrounded by n solvent molecules S, E_{S_0} is the electronic energy of the ion without any explicit solvent molecules, and E_S is the electronic energy of the uncoordinated solvent molecule.

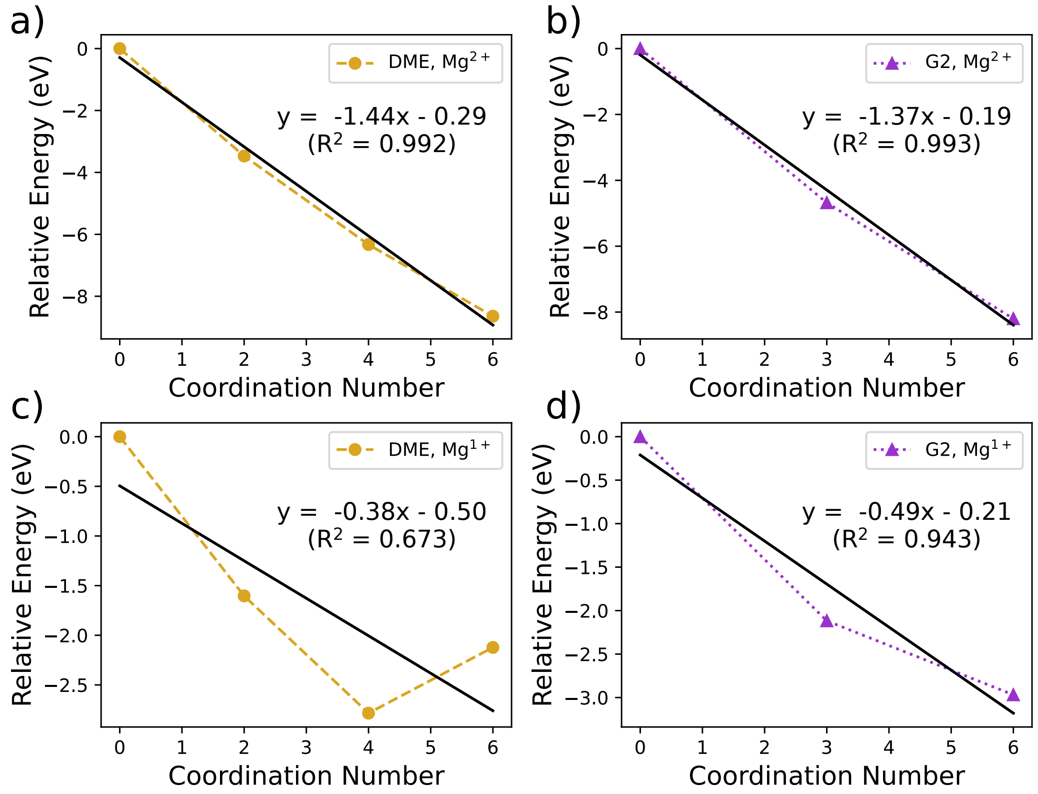

Figure S13: Relative stabilization of Mg ions (Mg^{2+}, a-b; Mg^{1+}, c-d) by dimethoxyethane (DME) (a, c) and G2 (b, d) in terms of electronic energy (in eV). Equations for lines of best fit (solid black lines) are provided.

By performing linear regression on the relative stabilization of the Mg ions as a function of the coordination number of the ion (Figure S13), we can find an effective solvation correction to apply during CRN construction. We note that the comparatively poor linear trend seen with Mg^{1+} ions reflects the preference of these ions for 5-fold coordination. The addition of a sixth coordinate bond is somewhat energetically unfavorable with DME as a solvent (the stabilization decreases when a third DME molecule, increasing the coordination number from 4 to 6, is added), and the addition of a second G2 molecule (increasing the coordination number from 3 to 6) leads to a much lower stabilization than the first G2.

Reduction Potentials

We considered redox potentials of Mg-coordinated species with and without full explicit solvent shells (Table S1). It can be seen that the inclusion of explicit solvent can dramatically affect the reduction potential, in some cases changing the calculated value by $\sim$ 2V. For the reduction of Mg-coordinated G2 ($M_1 \longrightarrow M_2$), the predictions of reduction potentials in implicit solvent and explicit solvent are in qualitative disagreement. In implicit solvent, Mg^{2+} is expected to reduce significantly before the Mg plating potential (0.64 V vs. Mg/Mg^{2+}), while when a full explicit solvent shell is included, the reduction potential is significantly below the plating potential (-1.32 V vs. Mg/Mg^{2+}).

Table S1: **Reduction potentials of Mg-coordinated species with and without explicit solvation. All potentials are reported referenced to the reduction potential of Mg. * = The reduced molecule dissociated during geometry optimization.**

Reaction	$E^{\circ}_{implicit}$ (V)	$E^{\circ}_{explicit}$ (V)
$M_1 \longrightarrow M_2$	0.64	-1.32
$M_4 \longrightarrow M_7$	3.51	3.26*
$M_6{}^{+}Mg^{2+} \longrightarrow M_8$	3.89	1.62

Without conducting additional dynamic simulations in the presence of explicit electrified interfaces, it is challenging to know in detail either the solvation behavior or the reduction behavior of Mg ions. In the main text, we report reduction potentials without explicit

solvent, as we expect that Mg ions will at least partially desolvate when in close proximity to an electrode or interphase surface. This is also supported by the preference of Mg^{1+} ions for lower coordination numbers than Mg^{2+} (see Solvation Correction above). Nonetheless, we expect nontrivial error in our reported reduction potentials.

Network Products

Of the 6,469 species included in our CRN, 85 are identified as network products. Of these 85, 14 have sufficiently low predicted solubility in G2 that they can be considered as possible gaseous products. The remaining 71 species are shown in Figures S14-S16.

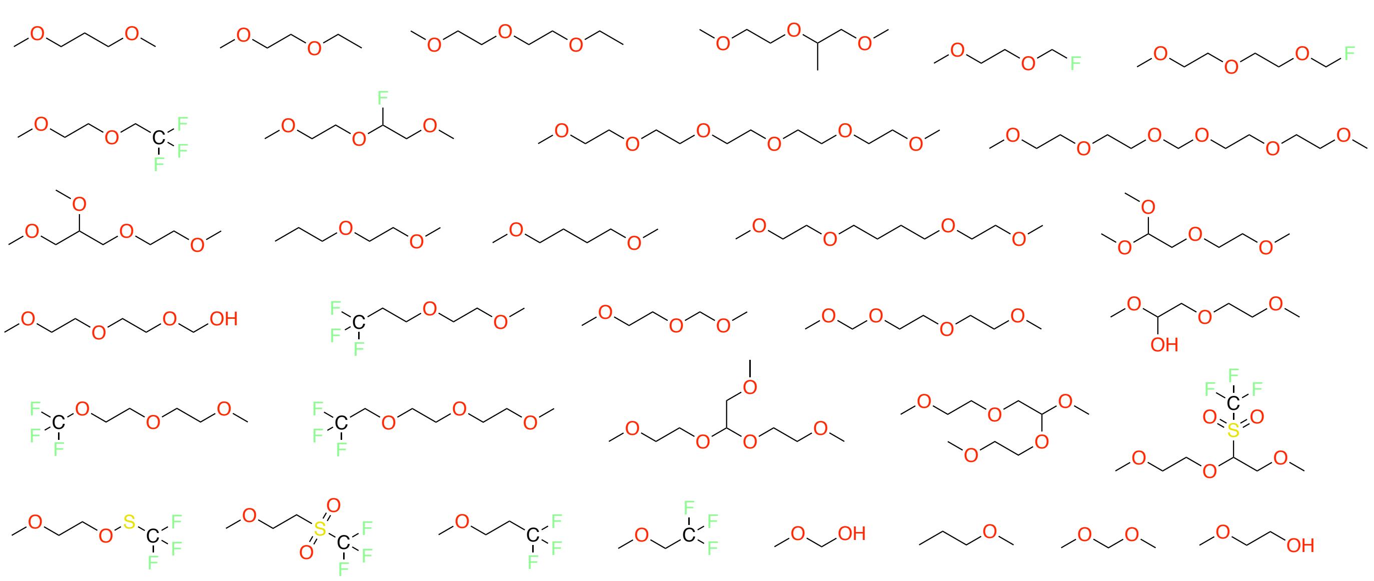

Figure S14: Oligomer and other chain hydrocarbon network products.

Thirty-three of the predicted products (Figure S14) have chain hydrocarbon backbones, in most cases with ether moieties. Many are oligomeric, including several branched oligomers. Considering these predicted network products alongside the propensity for G2 to decompose and form radical and anionic intermediates, we suggest that G2 should polymerize during MIB charging and SEI formation.

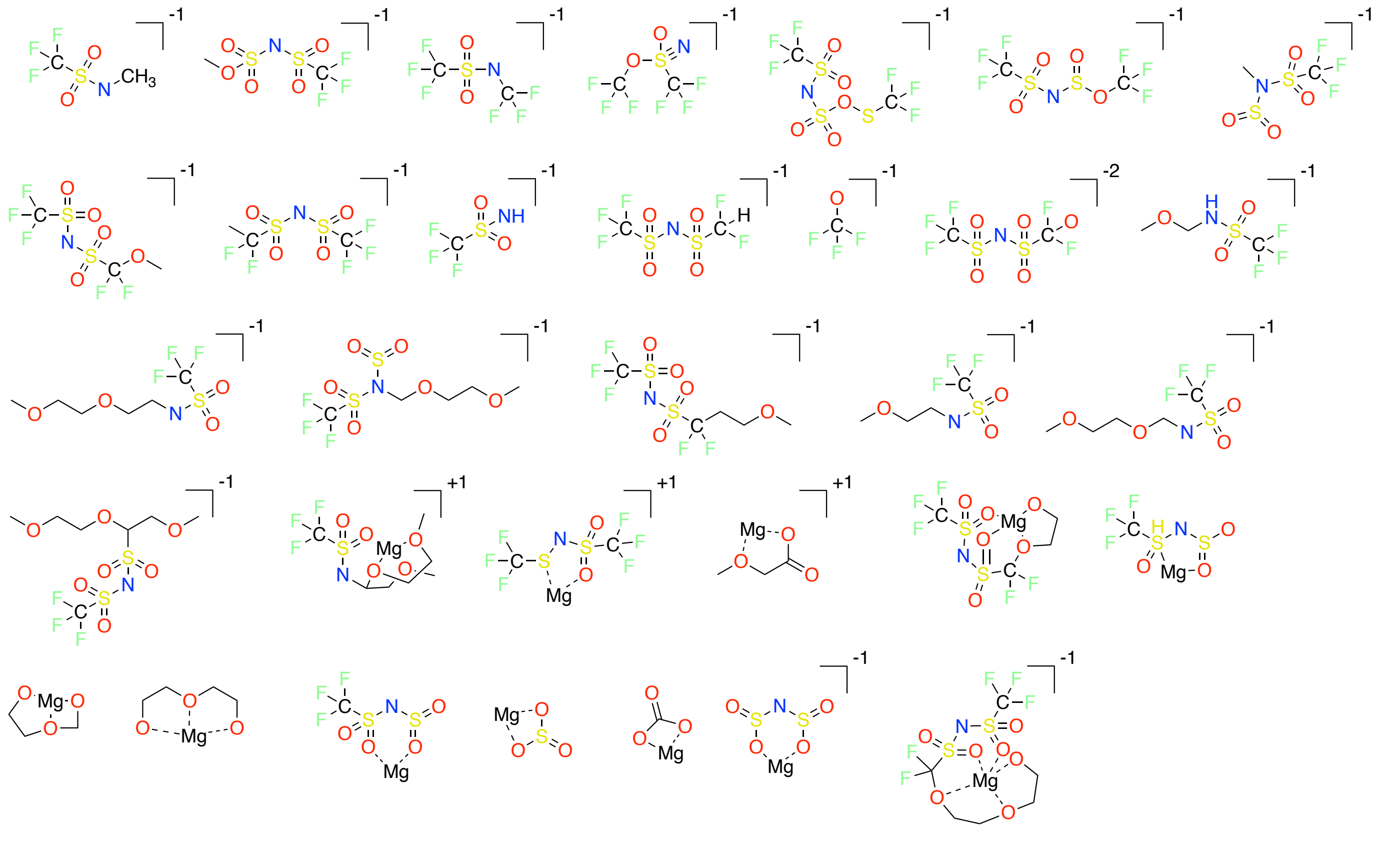

Figure S15: Salt network products.

An additional 32 network products contain non-magnesium ions or are magnesium salts (Figure S15). Without further experimental characterization, we cannot say much about most of these products and whether they are likely to emerge during MIB SEI formation. We suspect that many of these species are not likely stable and may continue to degrade (the decomposition products may be missing from the network). However, we do note that several salt products - namely $MgCO_3$[6,7] and $MgSO_3$[8] - have been observed by experimental spectroscopy.

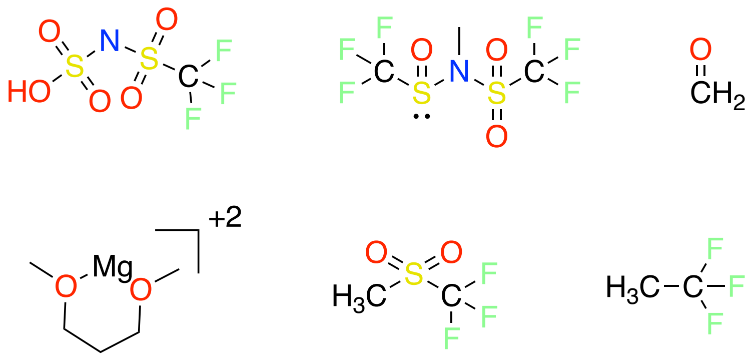

Figure S16: Other network products.

Scanning Electron Microscopy

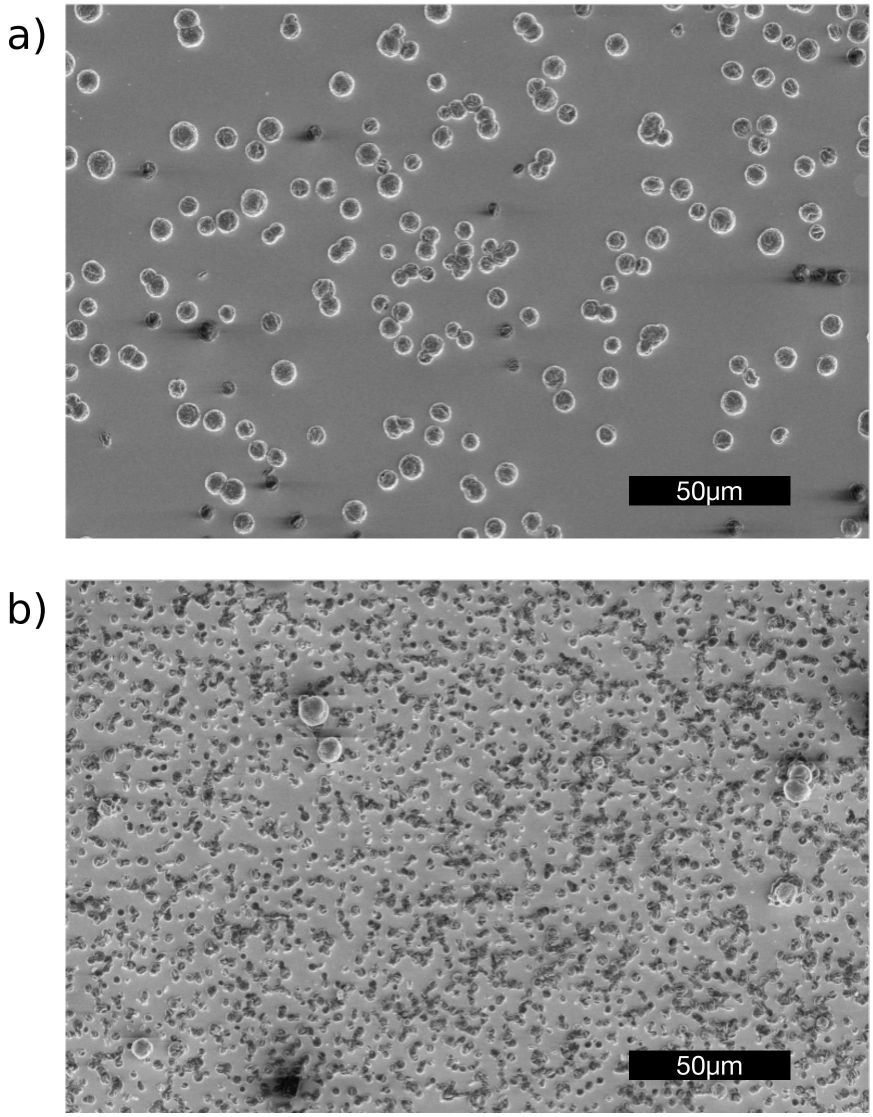

Figure S17: SEM images of remnant Mg and electrolyte ($TFSI^-$ and G2) decomposition products after 1 (a) and 10 (b) deposition and stripping cycles.

References

(1) Chai, J.-D.; Head-Gordon, M. Long-range corrected hybrid density functionals with damped atom–atom dispersion corrections. *Physical Chemistry Chemical Physics* **2008**, *10*, 6615–6620.

(2) Rappoport, D.; Furche, F. Property-optimized Gaussian basis sets for molecular response calculations. *The Journal of Chemical Physics* **2010**, *133*, 134105.

(3) Mardirossian, N.; Head-Gordon, M. B97X-V: A 10-parameter, range-separated hybrid, generalized gradient approximation density functional with nonlocal correlation, designed by a survival-of-the-fittest strategy. *Physical Chemistry Chemical Physics* **2014**, *16*, 9904–9924.

(4) Klamt, A. The COSMO and COSMO-RS solvation models. *WIREs Computational Molecular Science* **2011**, *1*, 699–709.

(5) Marenich, A. V.; Cramer, C. J.; Truhlar, D. G. Universal Solvation Model Based on Solute Electron Density and on a Continuum Model of the Solvent Defined by the Bulk Dielectric Constant and Atomic Surface Tensions. *The Journal of Physical Chemistry B* **2009**, *113*, 6378–6396.

(6) Yoo, H. D.; Han, S.-D.; Bolotin, I. L.; Nolis, G. M.; Bayliss, R. D.; Burrell, A. K.; Vaughey, J. T.; Cabana, J. Degradation Mechanisms of Magnesium Metal Anodes in Electrolytes Based on (CF3SO2)2N– at High Current Densities. *Langmuir* **2017**, *33*, 9398–9406.

(7) Gao, T.; Hou, S.; Huynh, K.; Wang, F.; Eidson, N.; Fan, X.; Han, F.; Luo, C.; Mao, M.; Li, X.; Wang, C. Existence of Solid Electrolyte Interphase in Mg Batteries: Mg/S Chemistry as an Example. *ACS Applied Materials & Interfaces* **2018**, *10*, 14767–14776.

(8) Jay, R.; Tomich, A. W.; Zhang, J.; Zhao, Y.; De Gorostiza, A.; Lavallo, V.; Guo, J. Comparative Study of Mg(CB11H12)2 and Mg(TFSI)2 at the Magnesium/Electrolyte Interface. *ACS Applied Materials & Interfaces* **2019**, *11*, 11414–11420.
